# Treating Opioid Use Disorder and Opioid Withdrawal in the Context of Fentanyl

**DOI:** 10.1146/annurev-clinpsy-081423-023518

**Published:** 2025-01-29

**Authors:** Suky Martinez, Jennifer D. Ellis, Cecilia L. Bergeria, Andrew S. Huhn, Kelly E. Dunn

**Affiliations:** Behavioral Pharmacology Research Unit, Department of Psychiatry and Behavioral Sciences, Johns Hopkins University School of Medicine, Baltimore, Maryland, USA

**Keywords:** opioid use disorder, opioid withdrawal syndrome, fentanyl, methadone, MOUD, heterogeneity, HiTOP

## Abstract

The opioid crisis, driven by illicitly manufactured fentanyl, presents significant challenges in treating opioid use disorder (OUD) and opioid withdrawal syndrome. Fentanyl is uniquely lethal due to its rapid onset and respiratory depressant effects, driving the surge in overdose deaths. This review examines the limitations of traditional diagnostic criteria like those of the *Diagnostic and Statistical Manual of Mental Disorders*, Fifth Edition, Text Revision (DSM-5-TR) and explores the potential of dimensional models such as the Hierarchical Taxonomy of Psychopathology (HiTOP) for a more nuanced understanding of OUD. Current treatments, including medications for OUD, are evaluated for efficacy in managing fentanyl-related OUD. Innovations in drug formulations and alternative induction methods are discussed to address the unique challenges posed by fentanyl. Psychotherapeutic and behavioral interventions, such as cognitive behavioral therapy and contingency management, are highlighted as crucial complements to pharmacotherapy. The review underscores the need for increased precision, comprehensive phenotyping, and advanced diagnostics to develop personalized treatment plans, all with the aim of improving patient outcomes and mitigating the societal impact of the opioid crisis.

## 1. INTRODUCTION TO OPIOID USE DISORDER

### 1.1. The Prevalence and Landscape of Opioid Use Disorder

Opioid use disorder (OUD) is a highly complex and heterogeneous disorder influenced by myriad genetic, environmental, and psychological factors. The disorder represents a profound public health crisis characterized by the chronic and relapsing nonmedical use of opioids, which include natural, semisynthetic, and synthetic substances such as heroin, fentanyl, and commercially produced pain relievers. In the United States, national samples suggest that up to 2.1 million noninstitutionalized civilian adults, or 0.9% of the population, meet criteria for OUD (Rhee & Rosenheck 2019). Globally, the burden of OUD is estimated to affect over 16 million individuals (Dydyk et al. 2024). Individuals with OUD face elevated risk for several opioid-related health consequences, including exposure to infectious diseases such as hepatitis C (Zibbell et al. 2018), human immunodeficiency virus (Strathdee et al. 2010), endocarditis (Figgatt et al. 2024), and methicillin-resistant *Staphylococcus aureus* (Parikh et al. 2020) as well as development or exacerbation of sleep problems (Ellis et al. 2022) and general injury (Pandya et al. 2015).

As the US drug supply has been largely overtaken by nonmedical, illicitly manufactured fentanyl (IMF) that now increasingly includes xylazine and other potent adulterants (D’Orazio et al. 2023), the number of fatal and nonfatal overdoses has increased drastically (Butelman et al. 2023, Spencer et al. 2024). It is currently estimated that a fatal overdose occurs once every 5 min in the United States and that more than one million people have died from drug-related overdoses in the past decade (Ahmad et al. 2024). This rate is so profound that drug-related overdose deaths are directly contributing to declines in the average life expectancy in the United States (Woolf & Schoomaker 2019). Since 2016, drug-related overdose deaths have been driven primarily by exposure to IMF. A recent increase in co-use of IMF with stimulants is leading to further exacerbations in fatal overdose rates (Ciccarone 2021); patients report co-using stimulants to offset the heavy sedative effects of IMF (Fredericksen et al. 2024). The collateral impact of these trends cannot be understated; not only is OUD potentially fatal to persons using opioids but it also devastates their families (C.M. Jones et al. 2024) and imposes substantial societal costs, including health care expenses, loss of productivity, and increased criminal justice involvement (Dieleman et al. 2020, Miller & Hendrie 2008).

This review introduces core concepts related to OUD and its treatment strategies before discussing features that make IMF a uniquely challenging issue for patients and the OUD treatment community.

### 1.2. Opioid Use Disorder Diagnostic Criteria and Limitations

OUD is broadly characterized by the *Diagnostic and Statistical Manual of Mental Disorders*, Fifth Edition, Text Revision (DSM-5-TR) as opioid use that becomes difficult for individuals to control; defining features are a persistent craving for opioids and use patterns that interfere with fulfillment of social or occupational roles (Am. Psychiatr. Assoc. 2022). Sustained exposure to opioids, whether licit or illicit in nature, engenders opioid tolerance and a corresponding physical dependence wherein individuals require higher doses of opioids to achieve similar or diminishing effects. Once physical dependence develops, a prominent opioid withdrawal syndrome will emerge during periods of opioid abstinence. Opioid withdrawal is a cluster of observable signs and self-reported symptoms that emerge in individuals with physical dependence who undergo a period of opioid abstinence. Opioid withdrawal symptoms may initially be mild but become severe with repeated opioid exposure; they can include mood changes (e.g., anxiety, restlessness, craving), insomnia, gastrointestinal symptoms (e.g., nausea, vomiting, cramping, diarrhea), bone and muscle pain, sympathetic nervous system activation (e.g., tachycardia, hypertension, pupil dilation, sweating), and other symptoms (e.g., yawning, runny nose, watery eyes, goosebumps). Whereas individuals who use opioids generally report initiating use for a positive effect (e.g., euphoria, analgesia), many people attribute their continued use of opioids, despite incurring negative consequences, to a drive to avoid the onset of the aversive withdrawal syndrome (Martinez et al. 2022). As such, management and/or alleviation of withdrawal symptoms is a target of many OUD treatments (Pergolizzi et al. 2020).

While the DSM-5-TR provides a standardized framework for diagnosis, it is not without limitations. The categorical nature of DSM-5-TR diagnoses often fails to capture the complexity and variability of psychopathology, leading to issues such as diagnostic heterogeneity, high comorbidity, and modest interrater reliability (Clark et al. 2017, Markon et al. 2011, Regier et al. 2013). Moreover, the DSM-5-TR’s reliance on discrete diagnostic categories does not reflect the continuous nature of mental disorder symptoms, resulting in information loss and potentially inadequate treatment strategies (Kotov et al. 2021). The failure of traditional approaches, as exemplified by the DSM-5-TR, to adequately address the complexity and heterogeneity of OUD and other mental disorders underscores the necessity for alternative models to complement our current diagnostic approach (Conway et al. 2021).

### 1.3. A Dimensional Approach to Opioid Use Disorder

The emergence of dimensional models, such as the Hierarchical Taxonomy of Psychopathology (HiTOP) (Kotov et al. 2017) and the NIDA Phenotyping Assessment Battery (PhAB), provides an opportunity for a more nuanced understanding of OUD (Conway et al. 2019, Watts et al. 2023). HiTOP offers an empirically derived model that seeks to address the limitations of traditional nosologies (e.g., DSM-5-TR) by conceptualizing psychopathology, including OUD, as a spectrum of symptoms and behaviors organized hierarchically from narrow dimensions to broad spectra and superspectra (Conway et al. 2019). This model, grounded in quantitative data, aims to capture the full range of mental disorders and their interrelationships.

For example, within the context of OUD, HiTOP can be used to identify specific dimensions such as reward processing, negative emotionality, and executive dysfunction (Watts et al. 2023). These dimensions can be further grouped into broader spectra, including externalizing and internalizing behaviors (Conway et al. 2022). Rather than forcing symptoms into the rigid categories imposed by the DSM-5-TR, HiTOP facilitates a fluid and continuous representation of symptoms and their severities (Kotov et al. 2022). This model is hypothesized to more accurately reflect the variability observed in OUD, from mild cases with few symptoms to severe cases with extensive comorbidities and functional impairments. The application of this approach is particularly compelling as we delve deeper into the heterogeneity of opioid withdrawal syndrome and the craving components of OUD. By delineating specific dimensions and their interrelations, the introduction of HiTOP to the field may enhance our understanding of the underlying mechanisms and pathways in OUD, thereby potentially improving diagnostics, treatment, and patient outcomes.

### 1.4. Opioid Withdrawal Syndrome: A Critical Component of Opioid Use Disorder

Opioid withdrawal syndrome has garnered significant attention due to its pivotal role in maintaining opioid use in individuals with OUD. Opioid withdrawal is characterized by a complex array of subjective and physiological symptoms that manifest during an initial acute phase followed by a protracted phase (Dunn et al. 2023). Broadly speaking, the acute withdrawal syndrome typically peaks within a few days and resolves within 2 weeks, and the protracted syndrome (characterized by mood disorders, insomnia, and cravings) can persist for several months, complicating the clinical picture of OUD (Dunn et al. 2019, Himmelsbach 1941, Himmelsbach & Andrews 1943). Numerous studies have documented the individual variability that exists in opioid withdrawal symptomatology (Dunn et al. 2020a,b). The expression of these symptoms can be influenced by factors such as the type of opioid used and the duration of physical dependence as well as by individual-level characteristics, including sex/gender (Huhn et al. 2018). Notably, shorter-acting opioids, such as heroin, are recognized as inducing a more rapid onset and resolution of acute withdrawal symptoms compared to longer-acting opioids, such as methadone (Gossop & Strang 1991). However, the proliferation of IMF in the drug market in the 2010s has generated considerable debate regarding the potential unique withdrawal profile exhibited by individuals with fentanyl exposure (Bird et al. 2023, Jones et al. 2020, Uddin et al. 2021), underscoring the need for further research in this area.

Precise and accurate measurement and assessment of opioid withdrawal syndrome are essential for developing effective interventions. Over the past several decades, numerous observer-rated and self-report scales have been developed to assess withdrawal severity, including the commonly used observer-rated Clinical Opiate Withdrawal Scale (COWS) (Wesson & Ling 2003) and self-reported Subjective Opiate Withdrawal Scale (SOWS) (Handelsman et al. 1987). Contemporary opioid withdrawal syndrome instruments attempt to incorporate both subjective and objective measures, providing a more comprehensive assessment, whereas earlier scale development often focused on observable signs to mitigate concerns about symptom exaggeration (Dunn et al. 2023). A multifaceted approach that combines subjective and objective assessments of withdrawal symptoms is essential because successful management of these symptoms may increase the likelihood of reducing opioid use and improving patient outcomes (Dunn & Strain 2024). Effectively managing withdrawal symptoms may enable patients to more fully engage in other aspects of treatment, thereby leading to broader improvements in the management of OUD. Additionally, given the variability in symptom expression and the complex interplay of acute and protracted phases, more research is needed to refine the measurement tools. Understanding the individual variability in opioid withdrawal symptom expression and the overall complexity of OUD highlights the need for personalized treatment approaches, which can substantially improve outcomes and quality of life for individuals diagnosed with this disorder.

## 2. RATIONALE AND INTRODUCTION TO MEDICATIONS FOR OPIOID USE DISORDER

### 2.1. Neurobiology and Mechanisms of Opioid Use Disorder

One of the prevailing neurobiological theories regarding the etiology of OUD posits that it is fundamentally a brain disorder marked by significant impairments and disruptions in the central nervous and stress systems (Strang et al. 2020). Opioid agonists exert their primary opioid-like effects through activity on the mu opioid receptors (MORs) located within the brain’s mesolimbic reward pathway, which includes key regions such as the ventral tegmental area (VTA) and the nucleus accumbens ( Jones et al. 2021). Upon binding to these receptors, opioids inhibit the release of gamma-aminobutyric acid, a neurotransmitter that typically suppresses dopamine release (Devine & Wise 1994, Lambert 2023), which consequently elevates dopamine levels and produces an intense euphoria that becomes associated with opioid exposure (Valentino & Volkow 2018). This pathway is well-characterized and central to the reinforcing effects of opioids, which drive the compulsion to continue drug use ( J.D. Jones et al. 2024, Uhl et al. 2019).

Over time, chronic opioid use induces neuroadaptive changes in the brain’s reward and stress systems (Strang et al. 2020). The brain compensates for the persistent presence of exogenously administered opioids by reducing the production of endogenous opioids and diminishing the sensitivity of opioid receptors (Lambert 2023); this lower sensitivity is a sign that physical dependence on opioids has developed. This neuroadaptation is the neurobiological basis of opioid tolerance, a hallmark feature of OUD that drives escalations in doses over time. As opioid use continues, the degree of physical dependence and tolerance deepens, and abstinence from exogenous opioids results in a profound opioid deficit that manifests as opioid withdrawal syndrome. Chronic opioid use also interacts with the amygdala, a region integral to processing stress and negative emotions (Bali et al. 2015). This interaction creates an antireward state characterized by heightened stress, anxiety, and dysphoria during withdrawal (Koob & Le Moal 2008). These neurobiological alterations underpin the compulsive nature of opioid use and the severe withdrawal symptoms experienced during cessation (Koob & Volkow 2016).

### 2.2. Opioid Withdrawal Symptoms and Medications

As described above, withdrawal symptoms are a direct consequence of the brain’s compensatory mechanisms to counteract excessive opioid activity. Once physical dependence has developed, any reduction or discontinuation of opioid use will cause the individual to experience a range of severe physical and psychological symptoms due to the overactive stress and antireward systems (Dunn et al. 2023). These symptoms include anxiety, restlessness, muscle pain, gastrointestinal distress, and autonomic hyperactivity (e.g., tachycardia, hypertension) (Dunn et al. 2023). Such symptoms often drive the individual to resume opioid use to alleviate discomfort, perpetuating the cycle of use (Koob & Le Moal 2008). Thus, to date, the most effective pharmacological treatments for OUD target the neurobiological mechanisms that alleviate withdrawal symptoms, reduce cravings, and restore normal brain function (Sofuoglu et al. 2019). Below, we briefly review the current medications for opioid use disorder (MOUDs) and some general treatment strategies.

#### 2.2.1. Opioid agonist treatment with methadone or buprenorphine.

The primary strategy to treat OUD in the United States is chronic administration of the long-acting opioids methadone and buprenorphine. Opioid agonist treatment is premised on the goal of managing opioid withdrawal syndrome and associated opioid cravings for patients. By eliminating the need to procure illicit opioid sources to suppress withdrawal, patients can focus their efforts on other therapeutic goals and/or receive wraparound services that can help them achieve sustained recovery. It should be emphasized, however, that agonist pharmacotherapies primarily manage the physiological aspects of withdrawal and that the behavioral components of OUD still benefit from treatment with additional supportive resources.

Agonist maintenance originated with methadone, a full agonist on the MOR that was developed as a long-acting opioid analgesic in 1946 and is currently classified as a Schedule II medication (Buchholz et al. 2023). Methadone was introduced as a treatment for OUD in the 1960s in response to a growing opioid crisis and a lack of effective treatment strategies. A series of landmark papers pioneered by Vincent Dole and Marie Nyswander demonstrated that individuals whose opioid use had been treated with daily methadone dosing were able to abstain from illicit use (Dole & Nyswander 1965), leading to rapid rollout of that public health strategy. The current methadone treatment infrastructure is highly codified in several federal statutes that impose strict requirements on this form of treatment, including dosing formulations and quantities, often requiring patients to visit specialized methadone clinics for daily visits to receive their medication (DHHS 2024).

Buprenorphine was developed in 1966 as a second long-acting opioid; it acts as a partial agonist on the mu, delta, and ORL1 opioid receptors and as an antagonist on the kappa opioid receptor, with a low ceiling on its MOR agonist activity (Strain & Gaddis 2023). Buprenorphine was proposed as an opioid treatment medication in the 1960s yet was not approved for that indication until 2002 (Campbell & Lovell 2012). Buprenorphine is classified as a Schedule III medication, which allows it to be prescribed directly from a provider’s office for the treatment of OUD (Fudala et al. 2003). This process initially required formal training and provider waivers from the Drug Enforcement Administration (DEA) to support office-based prescribing; however, a series of laws over the past decade has steadily eliminated these barriers, and buprenorphine is now accessible without these requirements in the Waiver Elimination (MAT Act). Innovation in buprenorphine delivery has also led to recent approvals of long-acting (1-week and 4-week) extended-release formulations of buprenorphine (Poliwoda et al. 2022). Buprenorphine primarily differs from methadone in its treatment delivery model; whereas methadone treatment programs are federally required to provide some level of behavioral health services, buprenorphine treatment programs are not (McKendrick et al. 2024). Thus, although buprenorphine is a more scalable treatment model than methadone, its treatment infrastructure primarily focuses on the physiological, not the behavioral, component to OUD.

Direct comparisons of methadone and buprenorphine reveal that they are roughly pharmacologically equal in helping to manage opioid withdrawal and reduce illicit behaviors, and differences tend to derive from variations in their treatment models. Retention in treatment is slightly better with methadone (Degenhardt et al. 2023) than with buprenorphine, yet buprenorphine, a partial MOR agonist, produces significantly less respiratory depression than methadone (and other full mu opioid agonists) and thus has greatly reduced overdose risk, thereby increasing its safety relative to that of methadone (Marteau et al. 2015). Maintenance on methadone and on buprenorphine are both associated with profound improvements in opioid-related consequences, including reductions in all-cause mortality (Santo et al. 2021) as well as criminal justice involvement and improvements in quality of life (Maremmani et al. 2007).

#### 2.2.2. Withdrawal mitigation with lofexidine.

As discussed, withdrawal management is a core component of treatment for OUD. Although methadone and buprenorphine are effective at managing withdrawal, their formal indication is for the treatment of OUD, a complex clinical condition of which opioid withdrawal is one component. In 2016, the nonopioid adrenergic agonist lofexidine was approved in the United States for the specific indication of opioid withdrawal (Fishman et al. 2019). Lofexidine has been used for opioid withdrawal management in Europe for several decades and is closely related to clonidine, an adrenergic agonist marketed for hypertension and used in the United States off-label for opioid withdrawal mitigation for the past 50 years (Gold et al. 1978). Lofexidine is not a scheduled drug and has no recognized risk for extramedical use or general addiction liability. Its withdrawal-relieving properties occur through its reduction of autonomic hyperactivity. Direct comparisons of lofexidine and clonidine suggest that lofexidine is equivalent or superior to clonidine in reducing withdrawal severity (Kuszmaul et al. 2020). Lofexidine is intended not as an independent treatment for OUD but rather as a treatment that can complement existing strategies.

#### 2.2.3. Preventing resumption of use with naltrexone.

Naltrexone is a long-acting MOR antagonist developed in 1963 that was originally approved as a treatment for alcohol use disorder before being approved in 1984 in oral formulation to prevent resumption of opioid use (Srivastava & Gold 2018). Because it is an antagonist, naltrexone is not scheduled and can be prescribed by any provider. Once consumed, oral naltrexone can block opioid agonist effects for up to 24 h (Lee et al. 1988), preventing patients who are seeking to achieve sustained recovery from experiencing positive opioid effects following use with once-daily naltrexone consumption. Because of its antagonist profile, great care must be exercised in initiating treatment with naltrexone to ensure that the patient has been fully withdrawn off opioids and has no recent agonist exposure; otherwise, naltrexone will precipitate a lengthy and painful opioid withdrawal syndrome (Sigmon et al. 2012). Recent approval of a long-acting injectable formulation of naltrexone (Vivitrol) now provides extended (30-day) protection against resumption of use. Despite being approved for several decades, naltrexone has not historically represented a large percentage of treatments for OUD because it requires a complete resolution of withdrawal before initiation, which can be a challenging process for many patients. Among those individuals who achieve complete opioid abstinence, naltrexone has demonstrated equivalent effectiveness to buprenorphine treatment (Lee et al. 2018) and strong retention in treatment (Zangiabadian et al. 2022).

### 2.3. Psychotherapeutic and Behavioral Interventions

In addition to the MOUDs described above, a variety of psychotherapeutic and behavioral interventions have demonstrated efficacy in the management of OUD. These psychological treatment modalities, diverse in their approaches, offer unique advantages, particularly when integrated with pharmacotherapies. An integrated treatment paradigm that combines pharmacotherapy with individualized psychotherapeutic interventions may offer greater opportunity to more comprehensively address the multifaceted nature of OUD. The integration of psychotherapeutic and behavioral interventions with MOUDs has shown effectiveness in enhancing patient outcomes in OUD treatment. Ongoing research is essential to further refine these integrated models and keep them accessible, cost-effective, and capable of addressing the complex needs of individuals with OUD. Given the high prevalence of comorbid mental disorders (e.g., depressive, anxiety, and personality disorders) among individuals with OUD (Santo et al. 2022), the integration of psychotherapeutic and behavioral interventions is critical for enhancing treatment outcomes and improving patient well-being. This comprehensive approach offers one of the most promising opportunities for sustained recovery and an improved quality of life for those affected by this chronic disorder. Here, we provide a concise review of the most effective psychotherapeutic and behavioral interventions for OUD.

#### 2.3.1. Cognitive behavioral therapy.

Among psychotherapeutic treatments, cognitive behavioral therapy (CBT) has garnered the most extensive research and clinical application for OUD (Morgenstern & Longabaugh 2000). CBT primarily targets the identification and modification of maladaptive thought patterns and behaviors associated with substance use (Magill et al. 2020, Ray et al. 2020). This treatment modality is administered by trained psychotherapists employing techniques such as cognitive restructuring, coping strategies, and relapse prevention (Magill et al. 2020). Notably, several studies indicate that CBT can be effectively delivered via technology-based platforms, including web-based modules, thereby enhancing treatment accessibility and adherence (Bickel et al. 2008, Christensen et al. 2014, Shi et al. 2019). However, the efficacy of CBT as a stand-alone intervention is often limited relative to its combined use with pharmacotherapy, underscoring the need for an integrated treatment model (Ray et al. 2020). For example, the combination of CBT with buprenorphine has been shown to significantly enhance treatment outcomes compared to buprenorphine alone (Gregory & Ellis 2020, Samples et al. 2022).

#### 2.3.2. Contingency management.

Contingency management (CM) is another effective behavioral intervention that has shown promise in OUD treatment. The core mechanism of CM is that positive reinforcement delivered contingently can be used to support behavior change, such as reductions in use or abstinence, adherence to treatment protocols, and achievement of other prosocial goals (Bolívar et al. 2021). This approach typically includes providing tangible rewards (e.g., vouchers, prizes), contingent on drug-free urine tests or other positive therapeutic goals (e.g., consistent attendance at therapy sessions), to reinforce therapeutic behaviors. Studies have shown support for the efficacy of CM in reducing illicit opioid use and improving treatment retention, particularly within methadone maintenance programs (Sofuoglu et al. 2019). Nonetheless, the implementation of CM faces challenges related to cost and sustainability, necessitating innovative strategies to maintain its effectiveness postintervention.

#### 2.3.3. Motivational interviewing.

Motivational interviewing (MI), another cost-effective intervention, is characterized by its directive, client-centered approach aimed at enhancing intrinsic motivation for change (Hettema et al. 2005). MI has demonstrated particular effectiveness in engaging patients who are otherwise ambivalent toward treatment (Sofuoglu et al. 2019). The intervention has shown prominent effects in acute care settings, such as emergency departments, where MI can facilitate immediate treatment engagement (Kaczorowski et al. 2020). However, the long-term impact of MI may require supplementary follow-up and ongoing support. General counseling and supportive psychotherapy are additional modalities that have been shown to improve medication adherence and address psychosocial issues unresolvable by medication alone (Dugosh et al. 2016). These therapies are especially beneficial in structured environments, such as supervised withdrawal or residential treatment programs, where they provide continuous support and improve overall treatment outcomes (Dugosh et al. 2016).

## 3. WHAT IS FENTANYL?

### 3.1. Historical Perspective

Fentanyl is an extremely selective synthetic agonist on the MOR that was prospectively engineered to be a highly lipophilic opioid that would rapidly penetrate the blood–brain barrier to provide quick onset of analgesia (Bista et al. 2015, Janssen 1962, Stanley 1992). Originally developed in 1959 and formally approved in the United States in 1968, fentanyl’s rapid onset and offset were ideal for surgical settings that valued the ability to easily titrate its profile of effects on and off when used during anesthesia. Fentanyl eventually developed a market for outpatient analgesic use, though its poor oral bioavailability necessitated the development of transdermal patches and lollipops for nonparenteral administration (Streisand et al. 1993).

### 3.2. Recent Use Patterns

Fentanyl can be synthesized relatively easily via a three- or four-step process, and slight variations in this process can result in production of various fentanyl analogs (Suh et al. 1998, Valdez et al. 2014). It may therefore be more accurate to refer to current use patterns as representing illicitly manufactured “fentanyls” versus fentanyl. There have been historical reports of extramedical fentanyl use, primarily beginning in the 1980s when it was sold under the name China White ( Jannetto et al. 2019). However, until recently its use patterns have been primarily extramedical use of commercially produced products that were fairly sporadic and clustered into geographic areas rather than widespread exposures (Berens et al. 1996; Kuhlman et al. 2003; Martin et al. 1991, 2006). In fact, a survey conducted with individuals using opioids in the mid-2000s concluded that fentanyl presented a low risk for extramedical use or general addiction liability because of its extreme potency and patient-level concerns regarding its potential toxicity (Cicero et al. 2010). However, in 2013 case reports began to emerge that IMF was being added to local heroin products (Algren et al. 2013), and by 2016 the presence of IMF in heroin products throughout the United States was ubiquitous. The adulteration of heroin with IMF has subsequently been attributed to various environmental and cultural factors, including the fact that IMF is a white powder that can easily be disguised as heroin, a growing cultural demand for heroin products combined with the fact that fentanyl can be synthesized more quickly than it takes for heroin to be naturally derived from a poppy plant, and the lower cost associated with production of a kilogram of IMF versus heroin ($3,500 versus $65,000, respectively) (DEA 2018, Frank & Pollack 2017). The adulteration of heroin with IMF happened very quickly, increasing >300% between 2014 and 2015 ( Jannetto et al. 2019), which prompted national alerts from regulatory agencies; levels would increase again during the COVID-19 pandemic. By 2018, the Centers for Disease Control and Prevention had declared fentanyl the deadliest drug in the United States.

### 3.3. Fentanyl’s Unique Pharmacology

Fentanyl binds to the MOR with a similar affinity as morphine (Ki = 1.17 nM) and has a similar half-life of effects (2–4 h), yet it is estimated to be 50–100 times more potent than morphine (Comer & Cahill 2019, Comer et al. 2008, Vearrier & Grundmann 2021). This may be due to differences in primary mu receptor binding sites (regions 6 and 7 for fentanyl versus regions 3 and 5 for morphine), the fact that fentanyl has relatively high selectivity for the mu receptor and low selectivity for the delta and kappa opioid receptors, and the fact that it is 1,000 times more lipophilic than morphine (Bista et al. 2015, Vearrier & Grundmann 2021). Fentanyl has many other characteristics that differentiate it from other opioids, including heroin. When administered intravenously, fentanyl rapidly crosses the blood–brain barrier and produces detectable subjective effects within 30–90 s (Baylon et al. 2000, Grell et al. 1970, Zacny et al. 1992), a feature that directly increases its reinforcing and euphoria-producing effects (Comer et al. 2009). Following this initial binding, fentanyl is rapidly redistributed into well-perfused peripheral organs (e.g., liver, lungs) and then secondarily into muscle and adipose tissue. For instance, an animal study demonstrated that within 5 min of intravenous administration, brain concentrations of fentanyl had already peaked and reduced by 90%, followed by redistribution (56% of the parent product) to peripheral muscles and then to peripheral adipose tissue (17% within 30 min) (Hug & Murphy 1981).

Repeated administration of fentanyl has also been shown to accumulate in muscle and adipose tissue (McClain & Hug 1980); though this has been shown in animals to increase sensitivity to subsequent fentanyl exposures, the clinical impact of this accumulation in humans has not been established (Novack et al. 1978). One potential consequence, however, is a secondary peaking phenomenon that is likely related to the peripheral sequestration of fentanyl. Evidence dating back to its original approval shows that some (but not all) individuals experience a phenomenon whereby sequestered fentanyl stores release fentanyl into the plasma and produce a secondary peak of fentanyl in the bloodstream (Mather 1983, Novack et al. 1978). These plasma levels can be clinically meaningful; a series of early reports found that some individuals who received fentanyl during surgery and showed no signs of opioid agonism immediately postsurgery experienced abrupt onsets of opioid-related consequences (including respiratory depression) that at times required antagonist administration several hours after their last fentanyl administration (Becker et al. 1976, Fung & Eisele 1980, McQuay et al. 1979, Stoeckel et al. 1979). Currently, the mechanism underlying the variability in the experience of secondary peaking is not understood.

Fentanyl is transformed by the P450 CYP3A4 enzyme into its primary metabolite norfentanyl, which is not currently recognized as conferring psychoactive effects (Tanaka et al. 2014, Wilde et al. 2019). Elimination occurs primarily via urine (76% of the parent product within 72 h) (Kuip et al. 2017, McClain & Hug 1980). One study conducted with individuals admitted to a residential treatment program reported that fentanyl and norfentanyl levels were detectable in urine samples for a mean 7.3 and 13.3 days, respectively (Huhn et al. 2020).

## 4. UNIQUE CONSIDERATIONS FOR PERSONS WITH FENTANYL EXPOSURE

Most data informing the consequences of fentanyl exposure derive from the medicinal fentanyl literature, which is characterized by doses of fentanyl that are orders of magnitude lower than current IMF exposure patterns. It is therefore currently unknown how these unique features of fentanyl may generalize to the IMF experience; however, a few notable trends, hypotheses, and extrapolations can be made.

### 4.1. Fentanyl-Induced Structural Changes That May Increase Risk of Fatal Overdose

IMF exposure has been the primary driver of opioid-related overdoses in the United States since 2016, and fentanyl has several features that likely contribute to its uniquely high lethality profile. First, fentanyl directly lowers oxygen consumption through its activity on the MOR, a phenomenon that has been well-characterized in surgical settings. For instance, a dose as small as 650 μg can decrease oxygenation to the point of respiratory depression in humans (Becker et al. 1976). In animals, fentanyl has been shown to produce deeper respiratory depression than either morphine or heroin, and opioid tolerance is also less protective against changes in respiration following fentanyl exposure relative to those comparators (Hill et al. 2020). An independent but related phenomenon is an onset of fentanyl-induced apneas and hyperapneas, which produce slow compensatory breathing patterns following frequent fentanyl administration (Algera et al. 2021, Haouzi et al. 2020).

In addition to altering respiration centrally, fentanyl exposure leads to structural changes that challenge respiration. The redistribution of fentanyl into peripheral muscles can produce (in some individuals) muscle rigidity that prevents the lungs from properly contracting. Also known as wooden chest syndrome (Torralva & Janowsky 2019), this muscle rigidity has been reliably observed when fentanyl is used in surgical settings, is most likely to occur when fentanyl is administered via rapid parenteral administration (consistent with how IMF is often administered), and is responsive to naloxone intervention, confirming its relationship with fentanyl exposure (Askgaard et al. 1977, Grell et al. 1970, Janis 1972, Rosenberg 1977). Muscle rigidity is not secondary to respiratory depression; rather, it represents an additional fentanyl-related risk for overdose that can emerge at doses of fentanyl lower than what is necessary to reduce respiration (Pergolizzi et al. 2021).

Fentanyl exposure can also cause a structural change in the larynx muscles known as a laryngospasm, or involuntary closure of vocal cords, that occludes all airflow to the lungs. Laryngospasms are transient but dose-dependent, and once they develop they may not be responsive to any current pharmacological interventions aside from high doses of the opioid antagonist naloxone. Though the presence of muscle rigidity and laryngospasms has not been definitely identified following IMF exposure, data from overdose reversal sites indicate that 30.7% of overdoses were characterized by muscle rigidity (often in the jaw or fingers, which directly impaired ambulatory interventions) (Kinshella et al. 2018), and there is a growing call to make oxygen available for persons experiencing overdose (perhaps for these reasons).

**Figure 1:**
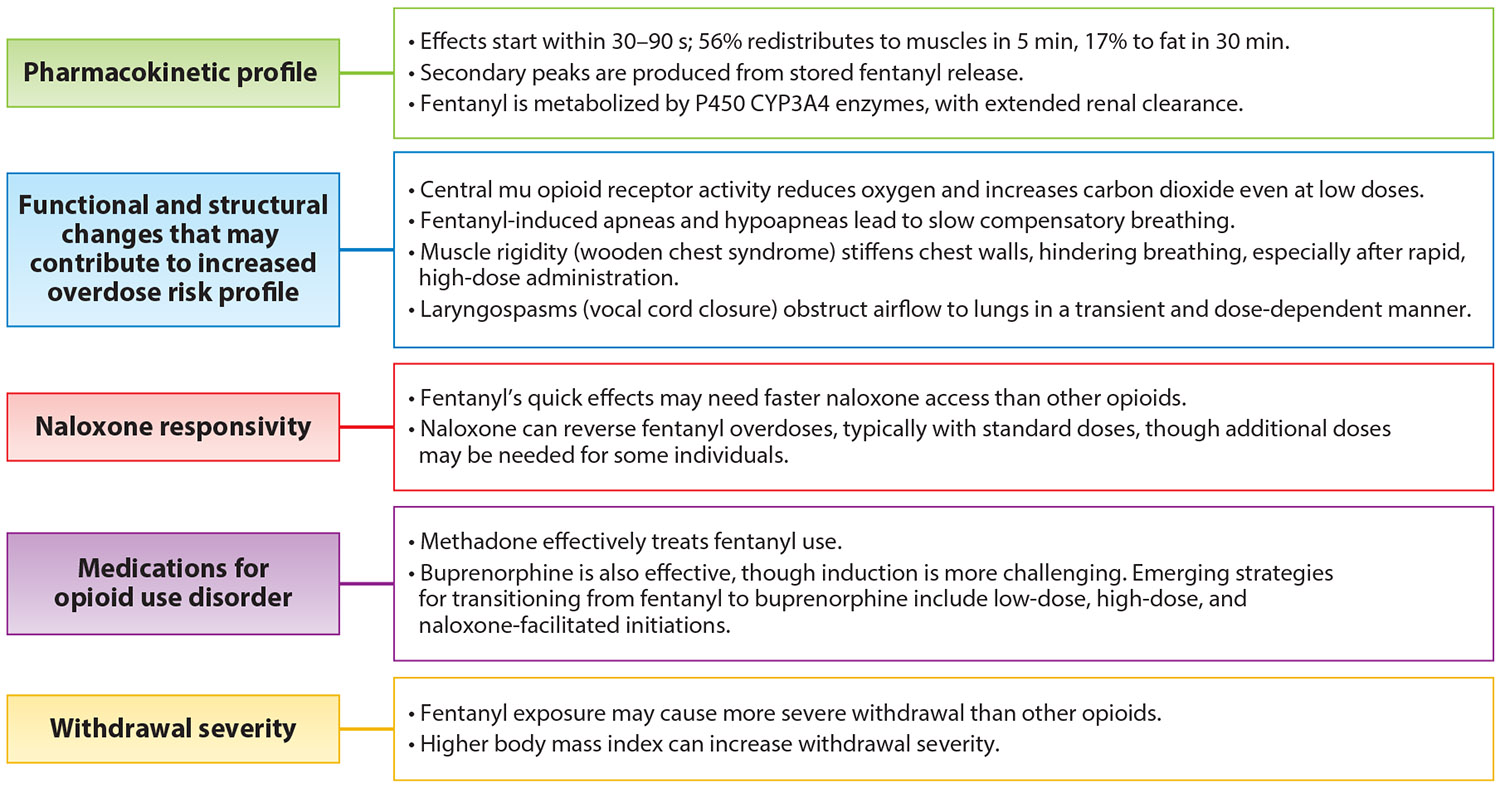
Unique features of fentanyl. Note that most evidence regarding the pharmacokinetic profile is from lower medicinal fentanyl doses, not higher illicit doses.

In summary, fentanyl’s significantly higher potency compared to that of morphine is attributed to its unique receptor binding and unique lipophilicity, facilitating rapid onset and redistribution within the body (Figure 1). As described, these pharmacokinetic properties can lead to delayed opioid effects and severe respiratory complications, including wooden chest syndrome and laryngospasms, thereby escalating the risk of fatal overdose. Although much of our understanding of these effects is derived from medicinal use, illicit fentanyl exposure introduces additional complexities and hazards. To better elucidate the distinct characteristics of fentanyl relative to other common opioids, Table 1 offers a comprehensive comparison, emphasizing differences in potency, pharmacokinetics, administration routes, overdose risk, medical applications, and regulatory status.

### 4.2. Naloxone to Reverse Overdose

There has been consistent debate as to whether a higher-potency opioid such as fentanyl will require a higher dose of the opioid antagonist naloxone to reverse respiratory depression than what has conventionally been effective for reversing overdoses related to morphine or heroin. There are a few points to consider. The first is that fentanyl’s fast onset of effects can result in a more rapid depression of respiration compared to other opioids, necessitating more immediate intervention with naloxone compared to other opioids (Darke & Duflou 2016). A second consideration is whether the potency and accumulation of fentanyl will alter the efficacy of naloxone. The data on this issue have been mixed. While it is clear that naloxone can displace fentanyl and reverse its agonist effects (Drummond et al. 1977), whether this can be achieved with a standard-dose administration (4 mg, intranasal) or whether higher naloxone doses are necessary is still being debated. One analysis of >26,000 individuals who received naloxone in an overdose setting found that only 34–57% of persons using fentanyl were responsive to a conventional naloxone dose (Moe et al. 2020), and a pharmacokinetic modeling study suggested that a high dose of naloxone may be required to adequately displace IMF from the receptor (Moss et al. 2020). However, a recent report of >9,800 overdose events in Kentucky revealed no significant change in the dose of naloxone required to successfully reverse an overdose during the period when IMF replaced the local heroin product (Rock et al. 2024). These data are further supported by observations during a human laboratory trial ( Jones et al. 2020) and review of emergency department records that found no relationship between IMF exposure and naloxone responsivity (Carpenter et al. 2020). Ultimately these data demonstrate that opioid education and naloxone distribution programs that expand training and access to naloxone remain a first-line strategy for reducing fentanyl-related mortality.

### 4.3. Medications for Opioid Use Disorder

In the face of the protracted overdose crisis, there have been substantial public health efforts to scale up access to the MOUDs methadone and buprenorphine to treat IMF use and reduce risk of fatal overdose. Early reports raised concerns regarding the ability of these medications to adequately treat the deep physical dependence engendered by repeated IMF exposure. Evidence now seems clear that methadone, a full mu receptor agonist, can effectively manage IMF-related withdrawal and presents a valuable treatment strategy (Carroll 1994). However, methadone’s treatment model requires frequent in-person visits to a clinic and imposes tight restrictions on dosing parameters and counseling requirements that are not easily amenable to rapid scaling. Thus, the ability of methadone to fight the opioid crisis may be limited more by its delivery model than by its pharmacological effects. Some recent studies have begun to leverage the “72-hour rule,” which permits opioids such as methadone to be prescribed for OUD treatment outside of an opioid treatment program for up to 72 h, to develop rapid methadone titration techniques that may provide a pathway to scale methadone treatment access for persons with IMF exposure (Liu et al. 2024, Shahlapour et al. 2024, Skogrand et al. 2024).

In contrast to challenges with methadone scaling, buprenorphine can now be prescribed for the treatment of OUD by any physician (regardless of their DEA waiver status), making it the most highly scalable MOUD treatment model. Yet buprenorphine may face some pharmacological challenges in the treatment of IMF. The first challenge relates to the buprenorphine induction process. Because it is a partial agonist with a high affinity for the MOR and a low ceiling on its effects, buprenorphine induction has always required that patients abstain from opioids for >12 h and be experiencing mild to moderate opioid withdrawal to initiate buprenorphine induction in a way that would not precipitate an acute opioid withdrawal syndrome ( Johnson 2003). Shortly after heroin began to be replaced with IMF, reports began to emerge that the conventional buprenorphine induction process was leading to a buprenorphine-precipitated opioid withdrawal syndrome in individuals for whom extended abstinence (>48–72 h) from fentanyl could be verified (Antoine et al. 2021, Varshneya et al. 2022). The mechanism underlying this precipitated withdrawal is unclear; it may be attributable to the fact that buprenorphine is approximately 1.7 times more potent than fentanyl and able to rapidly and thoroughly (>90%) displace fentanyl from the MOR (Boas & Villiger 1985), or it could be due to the peripheral sequestration and extended renal clearance of IMF that may be still be physiologically active.

At this time, there is no consensus method for transitioning patients from IMF to buprenorphine. Three primary strategies have been reported. The first strategy is the low-dose method (also referred to as the Bernese method), a technique developed to facilitate transition of patients from the long-acting full opioid agonist methadone to buprenorphine (Soyka 2021, Vogel et al. 2016). This strategy is premised on the notion that fentanyl is functionally a long-acting opioid with a short subjective profile of effects. The second strategy is the rapid administration of high-dose buprenorphine with no titration period (Weimer et al. 2023). This strategy is premised on the notion that emergent withdrawal following buprenorphine dosing in the context of IMF is not precipitated withdrawal but rather uncontrolled/unmanaged spontaneous withdrawal derived from the deep physical dependence produced by IMF, which can be offset by high doses of buprenorphine. The final approach, which has only been outlined in a few case reports, consists of self-administration of naloxone followed by rapid administration of high-dose buprenorphine. This strategy is premised on the notion that withdrawal during buprenorphine induction is a consequence of buprenorphine’s slow displacement of IMF, combined with its low ceiling of agonist effects, and that displacement of IMF can be more rapidly facilitated with naloxone and then quickly treated with buprenorphine saturation. At the time of this review, there is limited empirical evidence supporting each of these methods, and there have been no direct empirical comparisons to indicate which method may be the most effective. One additional approach that is emerging is the concept of “bridging” patients with short-acting opioids for a brief period (72 h) to effectively wash out acute IMF exposure before proceeding with a more traditional buprenorphine induction process (Thakrar et al. 2023).

Importantly, however, the experience of enhanced buprenorphine-precipitated withdrawal in the context of fentanyl has not been universally reported. For instance, one report of 1,200 patients participating in a multisite study of buprenorphine inductions in emergency departments found only 9 instances of buprenorphine-precipitated withdrawal, calling into question the authenticity of this concern (D’Onofrio et al. 2023). At the time of this review, the authenticity and mechanisms underlying buprenorphine-precipitated withdrawal in individuals with confirmed IMF abstinence are still unclear. Since buprenorphine is the most scalable MOUD treatment model, research that elucidates the prevalence of this interaction and its underlying mechanisms is paramount to patient care.

Finally, concerns had been initially raised that buprenorphine’s partial agonism would prevent it from adequately treating the deep physical dependence engendered by frequent IMF exposure. Thankfully, converging data have now reliably confirmed that buprenorphine remains an effective MOUD for persons with IMF exposure, particularly when administered at doses >24 mg/day (Chambers et al. 2023, Wakeman et al. 2019) or in a long-acting (versus sublingual) formulation (Nunes et al. 2024).

### 4.4. Illicitly Manufactured Fentanyl–Induced Differences in Opioid Withdrawal Severity

The enhanced potency and unique pharmacokinetic profile of IMF may also be evident in opioid withdrawal expression. Data to inform this question are only just emerging from quasi-experimental analyses generally derived from treatment initiation settings. One report found that individuals who presented to treatment admission with recent IMF exposure did not demonstrate a more severe form of opioid withdrawal in response to administration of the opioid antagonist naloxone relative to persons presenting with non-IMF opioid exposure ( Jones et al. 2020), a procedure that generally portends an individual’s spontaneous withdrawal syndrome severity (Dunn et al. 2017). A second report of individuals entering a residential trial found that recent fentanyl exposure was associated with more severe opioid withdrawal despite patients receiving consistent administration of morphine to reduce breakthrough symptoms. Moreover, withdrawal severity in that study was not meaningfully related to self-reported fentanyl exposure levels but was positively associated with higher body mass indices (BMIs), and persons with higher BMIs also demonstrated slower fentanyl clearance and reported more severe opioid withdrawal relative to individuals with lower BMIs (Luba et al. 2023). These data suggest that peripheral accumulation may contribute to fentanyl withdrawal expression. However, interpretation was limited due to the majority of participants in this trial (79%) having had recent fentanyl exposure.

A recent study (Sharma et al. 2024) provides a quasi-experimental comparison of withdrawal before and after infiltration of IMF into the local drug market. This study compared participants who were enrolled in a randomized controlled trial with confirmed IMF exposure to a historical control group who had completed a study with identical methods before the replacement of heroin with IMF in the local area. Both cohorts underwent identical opioid stabilization protocols wherein they received subcutaneous morphine (30 mg QID) for up to 11 days and completed several opioid withdrawal assessments per day. Opioid withdrawal was rated as significantly more severe on self-report and observer-rated measures for individuals in the IMF cohort relative to the historical control, and the two cohorts evinced distinctly different withdrawal severity time courses during the 7-day morphine stabilization period. Specifically, individuals with IMF exposure experienced nonlinear increases in withdrawal severity during the first 3 days followed by steady decreases in withdrawal over the remaining stabilization period, whereas individuals in the historical control demonstrated gradual, linear decreases in withdrawal each day. Moreover, withdrawal severity was elevated across all symptoms for persons with IMF exposure; no one symptom or array of symptoms drove the differences in peak withdrawal severity. However, there were also significant differences in heart rate and blood pressure across groups, which may support unique mechanisms involved in fentanyl-related withdrawal. Regional and stabilization approach differences likely play a role in how withdrawal may differ across individuals with and without fentanyl exposure.

### 4.5. Craving and the Complex Challenges Posed by Illicitly Manufactured Fentanyl

Understanding whether and how craving and related behaviors have shifted during the IMF era remains a significant challenge. A key obstacle in comparing across time (before and after IMF emergence) or between groups with and without IMF use is the absence of standardized, empirically validated measures of opioid craving (Kleykamp et al. 2019). However, the time course and pharmacological characteristics of fentanyl effects allow us to hypothesize how craving for fentanyl may function differently compared to craving for other opioids. Craving is generally characterized by anticipation or desire for either positive or negative reinforcing drug effects, a preoccupation with use, and the formation of plans to use (Kleykamp et al. 2019). Within the DSM-5-TR framework, craving has been proposed as a core feature in the maintenance of OUD. However, debate remains regarding the construct validity of craving and the challenges in effectively defining and operationalizing it within the context of OUD (Goodyear & Haass-Koffler 2020, Ray et al. 2013, Rosenberg 2009, Tiffany & Wray 2012). Furthermore, preliminary evidence suggests that opioid craving is a multidimensional construct with distinct components (Bergeria et al. 2021). Animal studies suggest that fentanyl and its analogs may intensify craving cycles compared to opioids like heroin or oxycodone due to their distinct pharmacological profile—marked by high potency, rapid onset, and short half-life (Maitland et al. 2024, Wheeler et al. 2023).

Given fentanyl’s short-acting nature, individuals who use IMF may experience more frequent and intense cravings as they are quickly confronted with either the desire for euphoric effects or the need to rapidly relieve withdrawal symptoms. This contrasts with other opioids—such as heroin, oxycodone, and methadone—that have longer durations of action due to their pharmacokinetic and pharmacodynamic properties. Compared to IMF, these opioids can provide individuals with extended periods of analgesia between doses, reducing the frequency of administration required to maintain the desired pharmacological effects and subjective experiences, such as euphoria. This distinction is further supported by a recent animal study that found that fentanyl engages neural circuits involved in both positive and negative reinforcement—specifically in the VTA and central amygdala—more intensely or with unique characteristics compared to other opioids (Chaudun et al. 2024). This suggests that individuals with chronic exposure to IMF may experience a unique pattern of craving, spending more time in a craving state—whether motivated by positive reinforcement (seeking euphoria) or negative reinforcement (avoiding withdrawal)—relative to individuals who use other forms of opioids.

The potential implications of this pattern could be significant. Individuals with chronic IMF exposure may experience heightened stress and increased drug-seeking behavior due to constant cycling between craving and withdrawal relief, further complicating their treatment plans. Consequently, chronic exposure to craving and stress may uniquely influence the treatment needs of individuals using IMF, highlighting the importance of rapid, accessible interventions that address both the intense euphoric desires and the frequent withdrawal symptoms they face. However, more empirical data and controlled human studies are needed to test this hypothesis.

Taken together, while current data on fentanyl-specific cravings remain limited, fentanyl’s distinct pharmacological profile suggests that cravings may play an especially intense and persistent role in the cycle of addiction. To fully understand how fentanyl-induced cravings may differ from those of other opioids in humans, comprehensive and systematic studies are crucial. Moreover, as mentioned above, the craving construct itself would greatly benefit from enhanced construct validity and more precise operationalization. Such improvements are fundamental to increase our understanding of the role that cravings play in the complexity of OUD.

## 5. DISCUSSION AND FUTURE DIRECTIONS

The current evidence strongly suggests that OUD is a multifaceted disorder with profound heterogeneity across numerous dimensions. OUD is known to vary widely regarding severity, types of opioids used, and co-occurring mental health disorders, making it difficult to develop a one-size-fits-all diagnostic and treatment approach. This reality presents a significant challenge and opportunity to better understand and develop effective interventions to help those affected by the disorder. As described in this review, there is a great need for a more nuanced understanding of how pharmacological, genetic, environmental, and psychological factors interact to influence the onset and progression of OUD. Studies indicate that individual differences in the neurobiology of addiction, such as variations in opioid receptor density and function, play a critical role in how OUD manifests and responds to treatment (Herlinger & Lingford-Hughes 2022). Furthermore, the high comorbidity of OUD with mental disorders like depression, anxiety, and personality disorders complicates the clinical picture and underscores the need for integrated treatment strategies that can address both substance use and mental health simultaneously (Santo et al. 2022, Sofuoglu et al. 2019).

As discussed in this review, in recent decades IMF has become pervasive within the illicit drug markets of North America (Ahmad et al. 2024, D’Orazio et al. 2023). IMF is now commonly detected not only in opioids but also in cocaine, methamphetamine, and counterfeit prescription medications, including those sold as sedatives and stimulants. While certain individuals with OUD may express a preference for fentanyl given its widespread and extended presence in the illicit drug supply (Hochstatter et al. 2022), individuals consuming black market substances may be unaware or have no control over the inclusion of fentanyl in their drugs (Martinez et al. 2021). Fentanyl’s unique pharmacology and high lethality are primarily attributed to its effects on oxygen consumption and respiration and have largely driven opioid-related overdoses in the United States since 2016. Fentanyl is known to lower oxygen consumption, producing deeper respiratory depression than morphine or heroin, and tolerance provides less protection against these respiratory changes. Fentanyl-induced changes in muscle rigidity (wooden chest syndrome), which impedes lung function and laryngospasms (involuntary closure of the vocal cords), also uniquely obstruct airflow to the lungs relative to other opioids. These effects, combined with data from overdose reversal sites reporting muscle rigidity in overdoses, highlight the need for both naloxone and oxygen availability during fentanyl-related overdoses.

However, it is important to note that most data on fentanyl’s physiological effects are derived from pharmaceutical-grade fentanyl, which is typically administered at significantly lower doses than the doses of IMF to which individuals are currently being exposed. Therefore, further research is needed to elucidate any potential differential effects of pharmaceutical fentanyl and IMF, especially when IMF is coadministered with other substances, to enhance the ecological validity of our current understanding. Additionally, although IMF has altered the reality and dynamics of the opioid crisis, currently available interventions have shown efficacy in managing OUD. For example, recent studies highlight the efficacy of buprenorphine for patients who present with IMF exposure. One study that conducted a post hoc analysis of a randomized clinical trial comparing extended-release buprenorphine to sublingual buprenorphine-naloxone found that the extended-release product significantly increased the proportion of opioid-negative urine samples in general and greater reductions in fentanyl exposure specifically relative to the sublingual product (Nunes et al. 2024). These benefits were also found in a second study that evaluated a 7-day injectable extended-release buprenorphine formulation for patients with minimal to mild opioid withdrawal (D’Onofrio et al. 2024). Together, these studies demonstrate the potential of extended-release buprenorphine to effectively manage OUD in individuals with fentanyl exposure, improving treatment outcomes and retention.

In the United States, available MOUDs have demonstrated efficacy in treating aspects of OUD; however, each treatment style has notable limitations and logistical challenges with regard to prescribing that affect their real-world effectiveness. For example, though methadone is highly effective in reducing opioid use and improving treatment retention, its use is often limited by regulatory barriers in the United States that restrict its availability to specialized clinics, hindering broader access for many patients (Sofuoglu et al. 2019). Buprenorphine can be prescribed in outpatient office-based settings, which improves its availability, yet buprenorphine treatment still faces challenges related to patient adherence and lack of behavioral support services (Bell & Strang 2020, Wakeman et al. 2020). Naltrexone, particularly in its extended-release formulations, shows promise in preventing resumption of opioid use but requires complete resolution of opioid withdrawal before initiation, which can be a barrier for many patients ( Jarvis et al. 2018, Johansson et al. 2006). To enhance the effectiveness of these existing pharmacotherapies, research has shown that combining them with psychological-behavioral interventions (e.g., CBT and CM), which can address some of the psychosocial aspects of OUD, may improve overall treatment outcomes (Bergman et al. 2019, Bolívar et al. 2021, Ray et al. 2020). Despite the availability of these pharmacological and psychological interventions, more work is needed to make significant improvements in OUD outcomes.

Studies have sought to address some of the challenges associated with the current treatments, especially as they relate to adherence and reducing return to opioid use. There has been a focus on the development of long-acting formulations of buprenorphine and naltrexone, such as injections or implants, which have enhanced these therapies (Krupitsky et al. 2019, Soyka & Franke 2021). Studies have shown that long-acting formulations of buprenorphine and naltrexone are effective (Krupitsky et al. 2013, Lofwall et al. 2018) but can be challenged with initiation and/or early discontinuation of treatment ( Jarvis et al. 2018, Lee et al. 2018). Within methadone treatment, much of the focus has involved expanding take-home methadone doses, which preliminary evidence suggests can be done safely and effectively (Dunn et al. 2021, Williams et al. 2023). In addition to pharmacotherapies, innovations in digital therapeutics, such as mobile apps that provide real-time support and monitoring and virtual reality therapies, have also shown some potential in improving treatment engagement and outcomes (Greenwald et al. 2024, Velez et al. 2021). Although these approaches are important to enhance the effectiveness of and access to currently approved treatments, more is needed to fully address the complex needs of individuals with OUD and to significantly reduce opioid-related mortality.

One of the most critical aspects needed to more comprehensively address these issues is greater advancements in the overall scientific approaches and methodologies in which OUD is investigated. Many of the constructs used to define OUD are only vaguely defined, display remarkable heterogeneity, and are inconsistently measured. For example, one of the primary targets of medication development, opioid withdrawal, is characterized by a profound heterogeneity of symptoms (Dunn et al. 2020a,b) that is compounded by a lack of precision in the manner by which opioid withdrawal is measured (Dunn & Strain 2024). Similarly, for another critical construct related to OUD, craving, current instruments do not capture all dimensions or the complexity of the construct (Bergeria et al. 2021). Taken together, it is clear that understanding OUD and developing more effective interventions relies on more refined definitions and measurement of our constructs. This will allow for greater specificity and precision in OUD research and facilitate a more nuanced understanding of the heterogeneity of the disorder.

Future research in OUD should specifically focus on multidimensional phenotyping guided by dimensional models such as HiTOP, which involves comprehensive assessments spanning behavioral, neurobiological, and genetic markers to identify distinct subtypes of OUD. Such detailed phenotyping can be used to support the development of personalized treatment plans tailored to individual risk profiles. The integration of neuroimaging techniques, such as functional magnetic resonance imaging and positron emission tomography scans, alongside improved diagnostics, could also provide additional insights into the structural and functional brain changes associated with OUD, helping to identify potential biomarkers for problematic use and/or resumption of use. Longitudinal studies that track individuals over time are also crucial to understanding the dynamic nature of OUD and the factors that contribute to long-term recovery or resumption of use. Advanced data analytics and machine learning can be leveraged to help identify latent patterns and/or predictors of treatment response, further enhancing the precision of OUD management. An important note is that there is also a lack of standardization of outcome measures in OUD treatment trials, in addition to a lack of consensus on optimal measures (Odom et al. 2023)—such as the definition of abstinence and the handling of missing data—which hinders the comparability of study results and evidence synthesis (Brandt et al. 2023, 2024).

In summary, OUD is a complex and heterogeneous disorder that requires a multifaceted approach to treatment and research. Current pharmacological treatments, while effective, face significant challenges in real-world application, necessitating the integration of psychological-behavioral interventions to enhance outcomes. Advances in long-acting formulations and digital therapeutics show promise in improving adherence and reducing relapse rates. However, further progress is needed to address the intricate needs of OUD patients fully. This includes refining scientific methodologies, emphasizing multidimensional phenotyping guided by dimensional models such as HiTOP, and leveraging neuroimaging and advanced analytics to develop personalized treatments. Standardizing outcome measures in OUD research is crucial for improving study comparability and evidence synthesis with the ultimate aim of reducing opioid-related mortality effectively.

## Figures and Tables

**Table 1:** Comparative analysis of fentanyl and other common opioids: potency, pharmacokinetics, administration routes, and risk profiles

Feature	Fentanyl (commercial and illicitly manufactured)	Morphine	Methadone	Oxycodone	Hydromorphone	Heroin
Potency	~50–100 times more potent than morphine	Standard reference opioid	Variable, but similar to morphine in equipotent doses	~1.5 times more potent than morphine	~5–7 times more potent than morphine	~2–3 times more potent than morphine
Onset of action	Rapid onset (minutes)	Intermediate onset (5–20 min)	Intermediate onset (5–20 min)	Intermediate onset (10–30 min)	Rapid onset (minutes)	Rapid onset (minutes)
Duration of action	Short (1–2 h)	Intermediate (3–4 h)	Intermediate (3–4 h)	Intermediate (3–6 h)	Intermediate (3–4 h)	Short (2–4 h)
Common administration routes	Commercial: transdermal, nasal, lozenges, intravenous IMF: intranasal, injectable, smoked	Oral, injectable	Oral, injectable	Oral, injectable	Oral, injectable	Injectable, intranasal, smoked
Overdose risk	High; very small dose can be fatal	Moderate; less potent, wider therapeutic index	Moderate; less potent, wider therapeutic index	Moderate; dose-dependent	High; similar to fentanyl	High; especially with variable purity
Medical uses	Commercial fentanyl: severe pain, anesthesia; no approved use of illicit fentanyl	Moderate to severe pain	Moderate to severe pain	Moderate to severe pain	Severe pain, especially in cases requiring high potency	No approved medical use in the United States; illicit
Regulation	Commercial fentanyl is regulated as a Schedule II controlled substance. Note that current fentanyl trends involve illicitly manufactured products.	Schedule II controlled substance	Schedule II controlled substance	Schedule II controlled substance	Schedule II controlled substance	Schedule I controlled substance

Abbreviation: IMF, illicitly manufactured fentanyl.

## SUMMARY POINTS

1. Fentanyl is significantly more potent than traditional opioids like morphine, and this higher potency contributes to its lethality, particularly through rapid respiratory depression. The use of illicitly manufactured fentanyl (IMF) in the drug supply has driven a surge in overdose deaths since 2016.
2. Traditional diagnostic frameworks, such as that of the Diagnostic and Statistical Manual of Mental Disorders, Fifth Edition, Text Revision (DSM-5-TR), may not capture the full complexity of opioid use disorder (OUD), particularly in the context of IMF use. OUD frequently co-occurs with other mental disorders, such as anxiety and depression, complicating treatment outcomes. Dimensional models like the Hierarchical Taxonomy of Psychopathology (HiTOP) offer more nuanced diagnostic tools for understanding the heterogeneity of OUD.
3. Opioid withdrawal is a major challenge in managing OUD; symptoms range from mood disturbances to severe physiological effects. Fentanyl’s unique properties may cause more severe withdrawal, and current tools that measure observed and self-reported withdrawal may need refinement for better accuracy in measuring these symptoms.
4. Methadone and buprenorphine remain the primary medications for OUD. Some studies indicate that buprenorphine doses greater than 24 mg daily are effective in treating fentanyl-related OUD. However, buprenorphine-precipitated withdrawal can occur, making induction challenging in patients exposed to fentanyl.
5. Innovations in buprenorphine induction, such as the Bernese method or high-dose administration, aim to address challenges related to fentanyl-induced withdrawal. Extended-release formulations of medications like buprenorphine show promise in improving adherence and reducing relapse.
6. Cognitive behavioral therapy (CBT) and contingency management (CM) are effective complementary treatments to pharmacotherapy in managing OUD. Integrated treatment models combining pharmacological and behavioral approaches show better outcomes than stand-alone treatments.
7. Despite concerns regarding the potency of fentanyl, recent evidence suggests that standard doses of naloxone remain effective in reversing fentanyl-induced respiratory depression. However, the rapid onset of fentanyl-related overdoses may necessitate quicker administration and higher community awareness for effective intervention.

## FUTURE ISSUES

1. The heterogeneity of OUD and the varying responses to currently available interventions underscore the need for innovation in understanding and treating OUD. Advanced diagnostic tools, multidimensional phenotyping, and personalized treatment approaches may hold promise and lead to identification of novel mechanisms and intervention targets.
2. Future research should focus on advancing diagnostic models beyond categorical systems like that of the DSM-5-TR. Integrating dimensional approaches such as HiTOP could offer more precise and personalized diagnostics, particularly for individuals with IMF exposure and co-occurring mental disorders. Further empirical validation of these models in clinical settings is necessary to better capture the heterogeneity of OUD.
3. Despite growing evidence of more severe withdrawal profiles in individuals exposed to IMF, the underlying neurobiological mechanisms remain unclear. Research should focus on understanding individual variability in opioid withdrawal severity, particularly in relation to IMF, to enhance treatment precision and patient outcomes. Longitudinal studies that employ comprehensive and biomarker analysis could help elucidate the physiological basis of enhanced withdrawal severity, informing more targeted treatment interventions.
4. Emerging methods such as the low-dose (Bernese) method and high-dose buprenorphine administration show promise in addressing fentanyl-related withdrawal challenges. Comparative clinical trials are needed to determine the most effective and scalable induction strategies, ensuring safe and rapid transitions onto buprenorphine in patients with deep physical dependence on fentanyl.
5. Given the complexity of OUD, future treatment paradigms should prioritize the integration of pharmacological and psychotherapeutic interventions. Research should explore novel combinations of long-acting medications like extended-release buprenorphine with evidence-based behavioral therapies, such as CBT and CM, to enhance patient outcomes and reduce relapse rates.
6. While medications for OUD like methadone and buprenorphine are effective, there is a growing need for non-opioid-based therapies that can mitigate withdrawal symptoms and prevent relapse. Future research should focus on the development and clinical testing of novel pharmacological agents, such as neuropeptide-based and adrenergic agonist compounds, that target alternative pathways in OUD and withdrawal management.

## References

[R1] Ahmad F, Cisewski J, Rossen L, Sutton P. 2024. Provisional drug overdose death counts. Rep., Natl. Cent. Health Stat, Hyattsville, MD

[R2] Algera MH, Olofsen E, Moss L, Dobbins RL, Niesters M, 2021. Tolerance to opioid-induced respiratory depression in chronic high-dose opioid users: a model-based comparison with opioid-naïve individuals. Clin. Pharmacol. Ther 109(3):637–45 32865832 10.1002/cpt.2027 PMC7983936

[R3] Algren DA, Monteilh CP, Punja M, Schier JG, Belson M, 2013. Fentanyl-associated fatalities among illicit drug users in Wayne County, Michigan ( July 2005-May 2006). J. Med. Toxicol 9(1):106–15 23359211 10.1007/s13181-012-0285-4 PMC3576499

[R4] Am. Psychiatr. Assoc. 2022. Diagnostic and Statistical Manual of Mental Disorders. Washington, DC: Am. Psychiatr. Publ. 5th ed., text rev.

[R5] Antoine D, Huhn AS, Strain EC, Turner G, Jardot J, 2021. Method for successfully inducting individuals who use illicit fentanyl onto buprenorphine/naloxone. Am. J. Addict 30(1):83–87 32572978 10.1111/ajad.13069 PMC7755703

[R6] Askgaard B, Nilsson T, Ibler M, Jansen E, Hansen JB. 1977. Muscle tone under fentanyl-nitrous oxide anaesthesia measured with a transducer apparatus in cholecystectomy incisions. Acta Anaesthesiol. Scand 21(1):1–4 139072 10.1111/j.1399-6576.1977.tb01185.x

[R7] Bali A, Randhawa PK, Jaggi AS. 2015. Stress and opioids: role of opioids in modulating stress-related behavior and effect of stress on morphine conditioned place preference. Neurosci. Biobehav. Rev 51:138–50 25636946 10.1016/j.neubiorev.2014.12.018

[R8] Baylon GJ, Kaplan HL, Somer G, Busto UE, Sellers EM. 2000. Comparative abuse liability of intravenously administered remifentanil and fentanyl. J. Clin. Psychopharmacol 20(6):597–606 11106130 10.1097/00004714-200012000-00002

[R9] Becker LD, Paulson BA, Miller RD, Severinghaus JW, Eger EI. 1976. Biphasic respiratory depression after fentanyl—droperidol or fentanyl alone used to supplement nitrous oxide anesthesia. Anesthesiology 44(4):291–95 1259186 10.1097/00000542-197604000-00003

[R10] Bell J, Strang J. 2020. Medication treatment of opioid use disorder. Biol. Psychiatry 87(1):82–88 31420089 10.1016/j.biopsych.2019.06.020

[R11] Berens AIL, Voets AJ, Demedts P., 1996. Illicit fentanyl in Europe. Lancet 347(9011):1334–35 10.1016/s0140-6736(96)90981-2 8622530

[R12] Bergeria CL, Strickland JC, Huhn AS, Strain EC, Dunn KE., 2021. A preliminary examination of the multiple dimensions of opioid craving. Drug Alcohol Depend. 219:108473 33429294 10.1016/j.drugalcdep.2020.108473 PMC10041947

[R13] Bergman BG, Fallah-Sohy N, Hoffman LA, Kelly JF. 2019. Psychosocial approaches in the treatment of opioid use disorders. In Treating Opioid Addiction, ed. Kelly JF, Wakeman SE, pp. 109–38. Cham, Switz.: Springer

[R14] Bickel WK, Marsch LA, Buchhalter AR, Badger GJ. 2008. Computerized behavior therapy for opioid-dependent outpatients: a randomized controlled trial. Exp. Clin. Psychopharmacol 16(2):132–43 18489017 10.1037/1064-1297.16.2.132 PMC2746734

[R15] Bird HE, Huhn AS, Dunn KE. 2023. Fentanyl absorption, distribution, metabolism, and excretion: narrative review and clinical significance related to illicitly manufactured fentanyl. J. Addict. Med 17(5):503–8 37788600 10.1097/ADM.0000000000001185 PMC10593981

[R16] Bista SR, Haywood A, Hardy J, Lobb M, Tapuni A, Norris R. 2015. Protein binding of fentanyl and its metabolite nor-fentanyl in human plasma, albumin and alpha-1 acid glycoprotein. Xenobiotica 45(3):207–12 25314012 10.3109/00498254.2014.971093

[R17] Boas RA, Villiger JW. 1985. Clinical actions of fentanyl and buprenorphine: the significance of receptor binding. Br. J. Anaesth 57(2):192–96 2982388 10.1093/bja/57.2.192

[R18] Bolívar HA, Klemperer EM, Coleman SRM, DeSarno M, Skelly JM, Higgins ST. 2021. Contingency management for patients receiving medication for opioid use disorder: a systematic review and meta-analysis. JAMA Psychiatry 78(10):1092–102 34347030 10.1001/jamapsychiatry.2021.1969 PMC8340014

[R19] Brandt L, Hu M-C, Nunes EV, Campbell ANC. 2023. Exploring the performance of during-treatment substance use outcome measures in predicting longer-term psychosocial functioning and post-treatment abstinence. Drug Alcohol Depend. 248:109918 37224673 10.1016/j.drugalcdep.2023.109918 PMC10680067

[R20] Brandt L, Odom GJ, Hu M, Castro C, Balise RR, CTN-0094 Team. 2024. Empirically contrasting urine drug screening-based opioid use disorder treatment outcome definitions. Addiction 119(7):1289–300 38616571 10.1111/add.16494 PMC12165254

[R21] Buchholz JR, Kalmoe M, Stamschror J, Saxon AJ. 2023. Methadone. In The Oxford Handbook of Opioids and Opioid Use Disorder, ed. Dunn KE, pp. 469–93. Oxford, UK: Oxford Univ. Press

[R22] Butelman ER, Huang Y, Epstein DH, Shaham Y, Goldstein RZ, 2023. Overdose mortality rates for opioids and stimulant drugs are substantially higher in men than in women: state-level analysis. Neuropsychopharmacology 48(11):1639–47 37316576 10.1038/s41386-023-01601-8 PMC10517130

[R23] Campbell ND, Lovell AM. 2012. The history of the development of buprenorphine as an addiction therapeutic. Ann. N.Y. Acad. Sci 1248(1):124–39 22256949 10.1111/j.1749-6632.2011.06352.x

[R24] Carpenter J, Murray BP, Atti S, Moran TP, Yancey A, Morgan B. 2020. Naloxone dosing after opioid overdose in the era of illicitly manufactured fentanyl. J. Med. Toxicol 16(1):41–48 31471760 10.1007/s13181-019-00735-w PMC6942078

[R25] Carroll KM. 1994. One-year follow-up of psychotherapy and pharmacotherapy for cocaine dependence. Arch. Gen. Psychiatry 51(12):989–97 7979888 10.1001/archpsyc.1994.03950120061010

[R26] Chambers LC, Hallowell BD, Zullo AR, Paiva TJ, Berk J, 2023. Buprenorphine dose and time to discontinuation among patients with opioid use disorder in the era of fentanyl. JAMA Netw. Open 6(9):e2334540 37721749 10.1001/jamanetworkopen.2023.34540 PMC10507490

[R27] Chaudun F, Python L, Liu Y, Hiver A, Cand J, 2024. Distinct μ-opioid ensembles trigger positive and negative fentanyl reinforcement. Nature 630(8015):141–48 38778097 10.1038/s41586-024-07440-x PMC11153127

[R28] Christensen DR, Landes RD, Jackson L, Marsch LA, Mancino MJ, 2014. Adding an Internet-delivered treatment to an efficacious treatment package for opioid dependence. J. Consult. Clin. Psychol 82(6):964–72 25090043 10.1037/a0037496 PMC4244262

[R29] Ciccarone D. 2021. The rise of illicit fentanyls, stimulants and the fourth wave of the opioid overdose crisis. Curr. Opin. Psychiatry 34(4):344–50 33965972 10.1097/YCO.0000000000000717 PMC8154745

[R30] Cicero TJ, Ellis MS, Paradis A, Ortbal Z. 2010. Determinants of fentanyl and other potent μ opioid agonist misuse in opioid-dependent individuals. Pharmacoepidemiol. Drug Saf 19(10):1057–63 20597128 10.1002/pds.1989 PMC2948061

[R31] Clark LA, Cuthbert B, Lewis-Fernández R, Narrow WE, Reed GM. 2017. Three approaches to understanding and classifying mental disorder: ICD-11, DSM-5, and the National Institute of Mental Health’s Research Domain Criteria (RDoC). Psychol. Sci. Public Interest 18(2):72–145 29211974 10.1177/1529100617727266

[R32] Comer SD, Ashworth JB, Sullivan MA, Vosburg SK, Saccone PA, Foltin RW. 2009. Relationship between rate of infusion and reinforcing strength of oxycodone in humans. J. Opioid Manag 5(4):203–12 19736900 10.5055/jom.2009.0022

[R33] Comer SD, Cahill CM. 2019. Fentanyl: receptor pharmacology, abuse potential, and implications for treatment. Neurosci. Biobehav. Rev 106:49–57 30528374 10.1016/j.neubiorev.2018.12.005 PMC7233332

[R34] Comer SD, Sullivan MA, Whittington RA, Vosburg SK, Kowalczyk WJ. 2008. Abuse liability of prescription opioids compared to heroin in morphine-maintained heroin abusers. Neuropsychopharmacology 33(5):1179–91 17581533 10.1038/sj.npp.1301479 PMC3787689

[R35] Conway CC, Forbes MK, Forbush KT, Fried EI, Hallquist MN, 2019. A hierarchical taxonomy of psychopathology can transform mental health research. Perspect. Psychol. Sci 14(3):419–36 30844330 10.1177/1745691618810696 PMC6497550

[R36] Conway CC, Forbes MK, South SC. 2022. A Hierarchical Taxonomy of Psychopathology (HiTOP) primer for mental health researchers. Clin. Psychol. Sci 10(2):236–58 35599839 10.1177/21677026211017834 PMC9122089

[R37] Conway CC, Krueger RF, Cicero DC, DeYoung CG, Eaton NR, 2021. Rethinking the diagnosis of mental disorders: data-driven psychological dimensions, not categories, as a framework for mental-health research, treatment, and training. Curr. Dir. Psychol. Sci 30(2):151–58

[R38] Darke S, Duflou J. 2016. The toxicology of heroin-related death: estimating survival times: heroin overdose. Addiction 111(9):1607–13 27082514 10.1111/add.13429

[R39] DEA (Drug Enforc. Adm.). 2018. 2016 Heroin Domestic Monitor Program. Rep., DEA, Springfield, VA

[R40] Degenhardt L, Clark B, Macpherson G, Leppan O, Nielsen S, 2023. Buprenorphine versus methadone for the treatment of opioid dependence: a systematic review and meta-analysis of randomised and observational studies. Lancet Psychiatry 10(6):386–402 37167985 10.1016/S2215-0366(23)00095-0

[R41] Devine D, Wise R. 1994. Self-administration of morphine, DAMGO, and DPDPE into the ventral tegmental area of rats. J. Neurosci 14(4):1978–84 8158252 10.1523/JNEUROSCI.14-04-01978.1994 PMC6577111

[R42] DHHS (Dep. Health Hum. Serv.). 2024. Medications for the treatment of opioid use disorder. Fed. Regist 89(23):7528–63

[R43] Dieleman JL, Cao J, Chapin A, Chen C, Li Z, 2020. US health care spending by payer and health condition, 1996–2016. JAMA 323(9):863–84 32125402 10.1001/jama.2020.0734 PMC7054840

[R44] Dole VP, Nyswander M. 1965. A medical treatment for diacetylmorphine (heroin) addiction: a clinical trial with methadone hydrochloride. JAMA 193(8):646–50 14321530 10.1001/jama.1965.03090080008002

[R45] D’Onofrio G, Hawk KF, Perrone J, Walsh SL, Lofwall MR, 2023. Incidence of precipitated withdrawal during a multisite emergency department-initiated buprenorphine clinical trial in the era of fentanyl. JAMA Netw. Open 6(3):e236108 36995717 10.1001/jamanetworkopen.2023.6108 PMC10064247

[R46] D’Onofrio G, Herring AA, Perrone J, Hawk K, Samuels EA, 2024. Extended-release 7-day injectable buprenorphine for patients with minimal to mild opioid withdrawal. JAMA Netw. Open 7(7):e2420702 38976265 10.1001/jamanetworkopen.2024.20702 PMC11231806

[R47] D’Orazio J, Nelson L, Perrone J, Wightman R, Haroz R. 2023. Xylazine adulteration of the heroin-fentanyl drug supply: a narrative review. Ann. Intern. Med 176(10):1370–76 37812779 10.7326/M23-2001

[R48] Drummond GB, Davie IT, Scott DB. 1977. Naloxone: dose-dependent antagonism of respiratory depression by fentanyl in anaesthetized patients. Br. J. Anaesth 49(2):151–54 319816 10.1093/bja/49.2.151

[R49] Dugosh K, Abraham A, Seymour B, McLoyd K, Chalk M, Festinger D. 2016. A systematic review on the use of psychosocial interventions in conjunction with medications for the treatment of opioid addiction. J. Addict. Med 10(2):93–103 26808307 10.1097/ADM.0000000000000193 PMC4795974

[R50] Dunn K, Bergeria C, Huhn AS, Strain EC. 2020a. Differences in patient-reported and observer-rated opioid withdrawal symptom etiology, time course, and relationship to clinical outcome. Drug Alcohol Depend. 215:108212 32781310 10.1016/j.drugalcdep.2020.108212

[R51] Dunn KE, Bergeria CL, Ware OD, Strain EC. 2023. The opioid withdrawal syndrome: presentation, measurement, and management. In The Oxford Handbook of Opioids and Opioid Use Disorder, ed. Dunn KE, pp. 194–233. Oxford, UK: Oxford Univ. Press

[R52] Dunn KE, Brooner RK, Stoller KB. 2021. Technology-assisted methadone take-home dosing for dispensing methadone to persons with opioid use disorder during the Covid-19 pandemic. J. Subst. Abuse Treat 121:108197 33357606 10.1016/j.jsat.2020.108197 PMC7834258

[R53] Dunn KE, Huhn AS, Bergeria CL, Gipson CD, Weerts EM. 2019. Non-opioid neurotransmitter systems that contribute to the opioid withdrawal syndrome: a review of preclinical and human evidence. J. Pharmacol. Exp. Ther 371(2):422–52 31391211 10.1124/jpet.119.258004 PMC6863456

[R54] Dunn KE, Strain EC. 2024. Establishing a research agenda for the study and assessment of opioid withdrawal. Lancet Psychiatry 11(7):566–72 38521089 10.1016/S2215-0366(24)00068-3

[R55] Dunn KE, Tompkins DA, Bigelow GE, Strain EC. 2017. Efficacy of tramadol extended-release for opioid withdrawal: a randomized clinical trial. JAMA Psychiatry 74(9):885–93 28700791 10.1001/jamapsychiatry.2017.1838 PMC5710234

[R56] Dunn KE, Weerts EM, Huhn AS, Schroeder JR, Tompkins DA, 2020b. Preliminary evidence of different and clinically meaningful opioid withdrawal phenotypes. Addict. Biol 25(1):e12680 30295400 10.1111/adb.12680 PMC6546557

[R57] Dydyk AM, Jain NK, Gupta M. 2024. Opioid use disorder. In StatPearls. Treasure Island, FL: StatPearls. https://www.ncbi.nlm.nih.gov/books/NBK553166/31985959

[R58] Ellis JD, Mayo JL, Gamaldo CE, Finan PH, Huhn AS. 2022. Worsening sleep quality across the lifespan and persistent sleep disturbances in persons with opioid use disorder. J. Clin. Sleep Med 18(2):587–95 34569924 10.5664/jcsm.9676 PMC8805005

[R59] Figgatt MC, Rosen DL, Chu VH, Wu L-T, Schranz AJ. 2024. Long-term risk of serious infections and mortality among patients surviving drug use-associated infective endocarditis. Clin. Infect. Dis 9(1):56–59 10.1093/cid/ciae214 PMC11259212 38642403

[R60] Fishman M, Tirado C, Alam D, Gullo K, Clinch T, Gorodetzky CW. 2019. Safety and efficacy of lofexidine for medically managed opioid withdrawal: a randomized controlled clinical trial. J. Addict. Med 13(3):169–76 30531234 10.1097/ADM.0000000000000474 PMC6541556

[R61] Frank RG, Pollack HA, 2017. Addressing the fentanyl threat to public health. N. Engl. J. Med 376(7):605–7 28199808 10.1056/NEJMp1615145

[R62] Fredericksen RJ, Baker R, Sibley A, Estadt AT, Colston D, 2024. Motivation and context of concurrent stimulant and opioid use among persons who use drugs in the rural United States: a multi-site qualitative inquiry. Harm Reduct. J 21(1):74 38561753 10.1186/s12954-024-00986-z PMC10985853

[R63] Fudala PJ, Bridge TP, Herbert S, Williford WO, Chiang CN, 2003. Office-based treatment of opiate addiction with a sublingual-tablet formulation of buprenorphine and naloxone. N. Engl. J. Med 349(10):949–58 12954743 10.1056/NEJMoa022164

[R64] Fung DL, Eisele JH. 1980. Fentanyl pharmacokinetics in awake volunteers. J. Clin. Pharmacol 20(11):652–58 7229112 10.1002/j.1552-4604.1980.tb01682.x

[R65] Gold MS, Redmond DE, Kleber HD, 1978. Clonidine in opiate withdrawal. Lancet 311(8070):929–30 10.1016/s0140-6736(78)90699-2 76860

[R66] Goodyear K, Haass-Koffler CL, 2020. Opioid craving in human laboratory settings: a review of the challenges and limitations. Neurotherapeutics 17(1):100–4 31650431 10.1007/s13311-019-00791-8 PMC7007448

[R67] Gossop M, Strang J. 1991. A comparison of the withdrawal responses of heroin and methadone addicts during detoxification. Br. J. Psychiatry 158(5):697–99 1860023 10.1192/bjp.158.5.697

[R68] Greenwald HJ, Berger A, Wilson RLH, Greenwald DJ, Lannon E, 2024. A pilot study of virtual reality for inpatients with opioid use disorder. Am. J. Addict 33(4):423–29 38430207 10.1111/ajad.13526

[R69] Gregory VL, Ellis RJB. 2020. Cognitive-behavioral therapy and buprenorphine for opioid use disorder: a systematic review and meta-analysis of randomized controlled trials. Am. J. Drug Alcohol Abuse 46(5):520–30 32960649 10.1080/00952990.2020.1780602

[R70] Grell FL, Koons RA, Denson JS. 1970. Fentanyl in anesthesia: a report of 500 cases. Anesth. Analg 49(4):523–32 5534663

[R71] Handelsman L, Cochrane KJ, Aronson MJ, Ness R, Rubinstein KJ, Kanof PD. 1987. Two new rating scales for opiate withdrawal. Am. J. Drug Alcohol Abuse 13(3):293–308 3687892 10.3109/00952998709001515

[R72] Haouzi P, Mellen N, McCann M, Sternick M, Guck D, Tubbs N. 2020. Evidence for the emergence of an opioid-resistant respiratory rhythm following fentanyl overdose. Respir. Physiol. Neurobiol 277:103428 32151709 10.1016/j.resp.2020.103428

[R73] Herlinger K, Lingford-Hughes A. 2022. Opioid use disorder and the brain: a clinical perspective. Addiction 117(2):495–505 34228373 10.1111/add.15636

[R74] Hettema J, Steele J, Miller WR. 2005. Motivational interviewing. Annu. Rev. Clin. Psychol 1:91–111 17716083 10.1146/annurev.clinpsy.1.102803.143833

[R75] Hill R, Santhakumar R, Dewey W, Kelly E, Henderson G. 2020. Fentanyl depression of respiration: comparison with heroin and morphine. Br. J. Pharmacol 177(2):254–65 31499594 10.1111/bph.14860 PMC6989952

[R76] Himmelsbach CK. 1941. The effects of certain chemical changes on the addiction characteristics of drugs of the morphine, codeine series. J. Pharmacol. Exp. Ther 71(1):42–48

[R77] Himmelsbach CK, Andrews HL. 1943. Studies on modification of the morphine abstinence syndrome by drugs. J. Pharmacol. Exp. Ther 77(1):17–23

[R78] Hochstatter KR, Terplan M, Mitchell SG, Schwartz RP, Dusek K, 2022. Characteristics and correlates of fentanyl preferences among people with opioid use disorder. Drug Alcohol Depend. 240:109630 36152404 10.1016/j.drugalcdep.2022.109630 PMC9616126

[R79] Hug CC, Murphy MR. 1981. Tissue redistribution of fentanyl and termination of its effects in rats. Anesthesiology 55(4):369–75 7294371 10.1097/00000542-198110000-00006

[R80] Huhn AS, Berry MS, Dunn KE. 2018. Systematic review of sex-based differences in opioid-based effects. Int. Rev. Psychiatry 30(5):107–16 30522374 10.1080/09540261.2018.1514295 PMC6551331

[R81] Huhn AS, Hobelmann JG, Oyler GA, Strain EC. 2020. Protracted renal clearance of fentanyl in persons with opioid use disorder. Drug Alcohol Depend. 214:108147 32650192 10.1016/j.drugalcdep.2020.108147 PMC7594258

[R82] Janis KM. 1972. Acute rigidity with small intravenous dose of Innovar: a case report. Anesth. Analg 51(3):375–76 5064016

[R83] Jannetto PJ, Helander A, Garg U, Janis GC, Goldberger B, Ketha H. 2019. The fentanyl epidemic and evolution of fentanyl analogs in the United States and the European Union. Clin. Chem 65(2):242–53 30305277 10.1373/clinchem.2017.281626

[R84] Janssen PAJ. 1962. A review of the chemical features associated with strong morphine-like activity. Br. J. Anaesth 34(4):260–68 14451235 10.1093/bja/34.4.260

[R85] Jarvis BP, Holtyn AF, Subramaniam S, Tompkins DA, Oga EA, 2018. Extended-release injectable naltrexone for opioid use disorder: a systematic review. Addiction 113(7):1188–209 29396985 10.1111/add.14180 PMC5993595

[R86] Johansson BA, Berglund M, Lindgren A. 2006. Efficacy of maintenance treatment with naltrexone for opioid dependence: a meta-analytical review. Addiction 101(4):491–503 16548929 10.1111/j.1360-0443.2006.01369.x

[R87] Johnson R 2003. Buprenorphine: how to use it right. Drug Alcohol Depend. 70(2):S59–77 12738351 10.1016/s0376-8716(03)00060-7

[R88] Jones CM, Zhang K, Han B, Guy GP, Losby J, 2024. Estimated number of children who lost a parent to drug overdose in the US from 2011 to 2021. JAMA Psychiatry 81(8):789–96 38717781 10.1001/jamapsychiatry.2024.0810 PMC11079787

[R89] Jones JD, Arout CA, Luba R, Murugesan D, Madera G, 2024. The influence of drug class on reward in substance use disorders. Pharmacol. Biochem. Behav 240:173771 38670466 10.1016/j.pbb.2024.173771 PMC11162950

[R90] Jones JD, Levin C, Mumtaz M, Comer S. 2021. Neurobiology of opioids. In The American Psychiatric Association Publishing Textbook of Substance Use Disorder Treatment, ed. Brady KT, Levin FR, Galanter M, Kleber HD, pp. 179–96. Washington, DC: Am. Psychiatr. Assoc. Publ.

[R91] Jones JD, Sherwin E, Martinez S, Comer SD. 2020. Naloxone-induced withdrawal in individuals with and without fentanyl-positive urine samples. Am. J. Addict 29(1):51–56 31782591 10.1111/ajad.12979 PMC6927546

[R92] Kaczorowski J, Bilodeau J, Orkin AM, Dong K, Daoust R, Kestler A. 2020. Emergency department–initiated interventions for patients with opioid use disorder: a systematic review. Acad. Emerg. Med 27(11):1173–82 32557932 10.1111/acem.14054

[R93] Kinshella M-LW, Gauthier T, Lysyshyn M. 2018. Rigidity, dyskinesia and other atypical overdose presentations observed at a supervised injection site, Vancouver, Canada. Harm Reduct. J 15(1):64 30577844 10.1186/s12954-018-0271-5 PMC6303894

[R94] Kleykamp BA, De Santis M, Dworkin RH, Huhn AS, Kampman KM, 2019. Craving and opioid use disorder: a scoping review. Drug Alcohol Depend. 205:107639 31683241 10.1016/j.drugalcdep.2019.107639

[R95] Koob GF, Le Moal M. 2008. Addiction and the brain antireward system. Annu. Rev. Psychol 59:29–53 18154498 10.1146/annurev.psych.59.103006.093548

[R96] Koob GF, Volkow ND. 2016. Neurobiology of addiction: a neurocircuitry analysis. Lancet Psychiatry 3(8):760–73 27475769 10.1016/S2215-0366(16)00104-8 PMC6135092

[R97] Kotov R, Cicero DC, Conway CC, DeYoung CG, Dombrovski A, 2022. The Hierarchical Taxonomy of Psychopathology (HiTOP) in psychiatric practice and research. Psychol. Med 52(9):1666–78 35650658 10.1017/S0033291722001301

[R98] Kotov R, Krueger RF, Watson D, Achenbach TM, Althoff RR, 2017. The Hierarchical Taxonomy of Psychopathology (HiTOP): a dimensional alternative to traditional nosologies. J. Abnorm. Psychol 126(4):454–77 28333488 10.1037/abn0000258

[R99] Kotov R, Krueger RF, Watson D, Cicero DC, Conway CC, 2021. The Hierarchical Taxonomy of Psychopathology (HiTOP): a quantitative nosology based on consensus of evidence. Annu. Rev. Clin. Psychol 17:83–108 33577350 10.1146/annurev-clinpsy-081219-093304

[R100] Krupitsky E, Blokhina E, Zvartau E, Verbitskaya E, Lioznov D, 2019. Slow-release naltrexone implant versus oral naltrexone for improving treatment outcomes in people with HIV who are addicted to opioids: a double-blind, placebo-controlled, randomised trial. Lancet HIV 6(4):e221–29 30880163 10.1016/S2352-3018(18)30362-X PMC6529232

[R101] Krupitsky E, Nunes EV, Ling W, Gastfriend DR, Memisoglu A, Silverman BL. 2013. Injectable extended- release naltrexone (XR-NTX) for opioid dependence: long-term safety and effectiveness. Addiction 108(9):1628–37 23701526 10.1111/add.12208

[R102] Kuhlman JJ, McCaulley R, Valouch TJ, Behonick GS. 2003. Fentanyl use, misuse, and abuse: a summary of 23 postmortem cases. J. Anal. Toxicol 27(7):499–504 14607006 10.1093/jat/27.7.499

[R103] Kuip EJM, Zandvliet ML, Koolen SLW, Mathijssen RHJ, Van Der Rijt CCD. 2017. A review of factors explaining variability in fentanyl pharmacokinetics; focus on implications for cancer patients. Br. J. Clin. Pharmacol 83(2):294–313 27619152 10.1111/bcp.13129 PMC5237702

[R104] Kuszmaul AK, Palmer EC, Frederick EK. 2020. Lofexidine versus clonidine for mitigation of opioid withdrawal symptoms: a systematic review. J. Am. Pharm. Assoc 60(1):145–52 10.1016/j.japh.2019.10.004 31791720

[R105] Lambert DG. 2023. Opioids and opioid receptors; understanding pharmacological mechanisms as a key to therapeutic advances and mitigation of the misuse crisis. BJA Open 6:100141 37588171 10.1016/j.bjao.2023.100141 PMC10430815

[R106] Lee JD, Nunes EV, Novo P, Bachrach K, Bailey GL, 2018. Comparative effectiveness of extended-release naltrexone versus buprenorphine-naloxone for opioid relapse prevention (X:BOT): a multicentre, open-label, randomised controlled trial. Lancet 391(10118):309–18 29150198 10.1016/S0140-6736(17)32812-X PMC5806119

[R107] Lee MC, Wagner HN, Tanada S, Frost JJ, Bice AN, Dannals RF. 1988. Duration of occupancy of opiate receptors by naltrexone. J. Nucl. Med 29(7):1207–11 2839637

[R108] Liu P, Chan B, Sokolski E, Patten A, Englander H. 2024. Piloting a hospital-based rapid methadone initiation protocol for fentanyl. J. Addict. Med 18(4):458–62 38832695 10.1097/ADM.0000000000001324 PMC11290994

[R109] Lofwall MR, Walsh SL, Nunes EV, Bailey GL, Sigmon SC, 2018. Weekly and monthly subcutaneous buprenorphine depot formulations versus daily sublingual buprenorphine with naloxone for treatment of opioid use disorder: a randomized clinical trial. JAMA Intern. Med 178(6):764–73 29799968 10.1001/jamainternmed.2018.1052 PMC6145749

[R110] Luba R, Jones J, Choi CJ, Comer S. 2023. Fentanyl withdrawal: understanding symptom severity and exploring the role of body mass index on withdrawal symptoms and clearance. Addiction 118(4):719–26 36444486 10.1111/add.16100 PMC9992259

[R111] Magill M, Tonigan JS, Kiluk B, Ray L, Walthers J, Carroll K. 2020. The search for mechanisms of cognitive behavioral therapy for alcohol or other drug use disorders: a systematic review. Behav. Res. Ther 131:103648 32474226 10.1016/j.brat.2020.103648 PMC7329023

[R112] Maitland AD, McGriff SA, Glatfelter GC, Schindler CW, Baumann MH. 2024. Reinforcing effects of fentanyl analogs found in illicit drug markets. Psychopharmacology 241:2375–83 38965085 10.1007/s00213-024-06641-6 PMC11513704

[R113] Maremmani I, Pani PP, Pacini M, Perugi G. 2007. Substance use and quality of life over 12 months among buprenorphine maintenance-treated and methadone maintenance-treated heroin-addicted patients. J. Subst. Abuse Treat 33(1):91–98 17588494 10.1016/j.jsat.2006.11.009

[R114] Markon KE, Chmielewski M, Miller CJ. 2011. The reliability and validity of discrete and continuous measures of psychopathology: a quantitative review. Psychol. Bull 137(5):856–79 21574681 10.1037/a0023678

[R115] Marteau D, McDonald R, Patel K. 2015. The relative risk of fatal poisoning by methadone or buprenorphine within the wider population of England and Wales. BMJ Open 5(5):e007629 10.1136/bmjopen-2015-007629 PMC4452747 26024998

[R116] Martin M, Hecker J, Clark R, Frye J, Jehle D, 1991. China White epidemic: an eastern United States emergency department experience. Ann. Emerg. Med 20(2):158–64 1996799 10.1016/s0196-0644(05)81216-8

[R117] Martin TL, Woodall KL, McLellan BA. 2006. Fentanyl-related deaths in Ontario, Canada: toxicological findings and circumstances of death in 112 cases (2002–2004). J. Anal. Toxicol 30(8):603–10 17132259 10.1093/jat/30.8.603

[R118] Martinez S, Brandt L, Comer SD, Levin FR, Jones JD. 2022. The subjective experience of heroin effects among individuals with chronic opioid use: revisiting reinforcement in an exploratory study. Addict. Neurosci 4:100034 36120106 10.1016/j.addicn.2022.100034 PMC9481059

[R119] Martinez S, Jones JD, Brandt L, Campbell ANC, Abbott R, Comer SD. 2021. The increasing prevalence of fentanyl: a urinalysis-based study among individuals with opioid use disorder in New York City. *Am. J. Addict.* 30(1):65–71 10.1111/ajad.13092 PMC7816517 32776640

[R120] Mather LE. 1983. Clinical pharmacokinetics of fentanyl and its newer derivatives. Clin. Pharmacokinet 8(5):422–46 6226471 10.2165/00003088-198308050-00004

[R121] McClain DA, Hug CC. 1980. Intravenous fentanyl kinetics. Clin. Pharmacol. Ther 28(1):106–14 7389247 10.1038/clpt.1980.138

[R122] McKendrick G, Stull SW, Sharma A, Dunn KE. 2024. Availability and opportunities for expansion of buprenorphine for the treatment of opioid use disorder. Semin. Neurol 44(4):419–29 38876459 10.1055/s-0044-1787569

[R123] McQuay HJ, Moore RA, Paterson GMC, Adams AP. 1979. Plasma fentanyl concentrations and clinical observations during and after operation. Br. J. Anaesth 51(6):543–50 465272 10.1093/bja/51.6.543

[R124] Miller T, Hendrie D. 2008. Substance abuse prevention dollars and cents: a cost-benefit analysis. Rep. (SMA) 07–4298, Subst. Abuse Ment. Health Serv. Adm., Rockville, MD

[R125] Moe J, Godwin J, Purssell R, O’Sullivan F, Hau JP, 2020. Naloxone dosing in the era of ultra-potent opioid overdoses: a systematic review. Can. J. Emerg. Med 22(2):178–86 10.1017/cem.2019.471 31955714

[R126] Morgenstern J, Longabaugh R. 2000. Cognitive-behavioral treatment for alcohol dependence: a review of evidence for its hypothesized mechanisms of action. Addiction 95(10):1475–90 11070524 10.1046/j.1360-0443.2000.951014753.x

[R127] Moss RB, Pryor MM, Baillie R, Kudrycki K, Friedrich C, 2020. Higher naloxone dosing in a quantitative systems pharmacology model that predicts naloxone-fentanyl competition at the opioid mu receptor level. PLOS ONE 15(6):e0234683 32544184 10.1371/journal.pone.0234683 PMC7297366

[R128] Novack GD, Bullock JL, Eisele JH. 1978. Fentanyl: cumulative effects and development of short-term tolerance. Neuropharmacology 17(1):77–82 652134 10.1016/0028-3908(78)90177-6

[R129] Nunes EV, Comer SD, Lofwall MR, Walsh SL, Peterson S, 2024. Extended-release injection versus sublingual buprenorphine for opioid use disorder with fentanyl use: a post hoc analysis of a randomized clinical trial. JAMA Netw. Open 7(6):e2417377 38916892 10.1001/jamanetworkopen.2024.17377 PMC11200143

[R130] Odom GJ, Brandt L, Castro C, Luo SX, Feaster DJ, 2023. Capturing drug use patterns at a glance: an *n*-ary word sufficient statistic for repeated univariate categorical values. PLOS ONE 18(9):e0291248 37682922 10.1371/journal.pone.0291248 PMC10490938

[R131] Pandya U, O’Mara MS, Wilson W, Opalek J, Lieber M. 2015. Impact of preexisting opioid use on injury mechanism, type, and outcome. J. Surg. Res 198(1):7–12 26088083 10.1016/j.jss.2015.05.033

[R132] Parikh MP, Octaria R, Kainer MA. 2020. Methicillin-resistant staphylococcus aureus bloodstream infections and injection drug use, Tennessee, USA, 2015–2017. Emerg. Infect. Dis 26(3):446–53 32091385 10.3201/eid2603.191408 PMC7045815

[R133] Pergolizzi JV, Raffa RB, Rosenblatt MH. 2020. Opioid withdrawal symptoms, a consequence of chronic opioid use and opioid use disorder: current understanding and approaches to management. J. Clin. Pharm. Ther 45(5):892–903 31986228 10.1111/jcpt.13114

[R134] Pergolizzi JV Jr., Webster LR, Vortsman E, LeQuang JA, Raffa RB. 2021. Wooden chest syndrome: the atypical pharmacology of fentanyl overdose. J. Clin. Pharm. Ther 46(6):1505–8 34240442 10.1111/jcpt.13484

[R135] Poliwoda S, Noor N, Jenkins JS, Stark CW, Steib M, 2022. Buprenorphine and its formulations: a comprehensive review. Health Psychol. Res. 10(3):37517 35999975 10.52965/001c.37517 PMC9392838

[R136] Ray LA, Courtney KE, Bacio G, MacKillop J. 2013. The assessment of craving in addiction research. In The Wiley-Blackwell Handbook of Addiction Psychopharmacology, ed. MacKillop J, de Wit H, pp. 345–80. Chichester, UK: Wiley

[R137] Ray LA, Meredith LR, Kiluk BD, Walthers J, Carroll KM, Magill M. 2020. Combined pharmacotherapy and cognitive behavioral therapy for adults with alcohol or substance use disorders: a systematic review and meta-analysis. JAMA Netw. Open 3(6):e208279 32558914 10.1001/jamanetworkopen.2020.8279 PMC7305524

[R138] Regier DA, Narrow WE, Clarke DE, Kraemer HC, Kuramoto SJ, 2013. DSM-5 field trials in the United States and Canada, Part II: test-retest reliability of selected categorical diagnoses. Am. J. Psychiatry 170(1):59–70 23111466 10.1176/appi.ajp.2012.12070999

[R139] Rhee TG, Rosenheck RA. 2019. Association of current and past opioid use disorders with health-related quality of life and employment among US adults. Drug Alcohol Depend. 199:122–28 31039486 10.1016/j.drugalcdep.2019.03.004 PMC6538934

[R140] Rock P, Slavova S, Westgate PM, Nakamura A, Walsh SL. 2024. Examination of naloxone dosing patterns for opioid overdose by emergency medical services in Kentucky during increased fentanyl use from 2018 to 2021. Drug Alcohol Depend. 255:111062 38157702 10.1016/j.drugalcdep.2023.111062 PMC11057324

[R141] Rosenberg H 2009. Clinical and laboratory assessment of the subjective experience of drug craving. Clin. Psychol. Rev 29(6):519–34 19577831 10.1016/j.cpr.2009.06.002

[R142] Rosenberg M 1977. Muscle rigidity with fentanyl: a case report. Anesth. Prog 24(2):50–52 274086 PMC2516049

[R143] Samples H, Williams AR, Crystal S, Olfson M. 2022. Psychosocial and behavioral therapy in conjunction with medication for opioid use disorder: patterns, predictors, and association with buprenorphine treatment outcomes. J. Subst. Abuse Treat 139:108774 35337716 10.1016/j.jsat.2022.108774 PMC9187597

[R144] Santo T, Campbell G, Gisev N, Martino-Burke D, Wilson J, 2022. Prevalence of mental disorders among people with opioid use disorder: a systematic review and meta-analysis. Drug Alcohol Depend. 238:109551 35797876 10.1016/j.drugalcdep.2022.109551

[R145] Santo T, Clark B, Hickman M, Grebely J, Campbell G, 2021. Association of opioid agonist treatment with all-cause mortality and specific causes of death among people with opioid dependence: a systematic review and meta-analysis. JAMA Psychiatry 78(9):979–93 34076676 10.1001/jamapsychiatry.2021.0976 PMC8173472

[R146] Shahlapour M, Singh S, Christine PJ, Laks J, Evans J, 2024. Novel uses of methadone under the “72-hour rule” to facilitate transitions of care and low-dose buprenorphine induction in an outpatient bridge clinic. J. Addict. Med 18(3):345–47 38329815 10.1097/ADM.0000000000001281

[R147] Sharma A, Dunn KE, Schmid-Doyle K, Dowell S, Kim N, 2024. Examining the severity and progression of illicitly manufactured fentanyl withdrawal: a quasi-experimental comparison. J. Addict. Med 10.1097/ADM.0000000000001395 PMC12429306 39591629

[R148] Shi JM, Henry SP, Dwy SL, Orazietti SA, Carroll KM. 2019. Randomized pilot trial of web-based cognitive- behavioral therapy adapted for use in office-based buprenorphine maintenance. Subst. Abuse 40(2):132–35 10.1080/08897077.2019.1569192 PMC6874094 30714880

[R149] Sigmon SC, Bisaga A, Nunes EV, O’Connor PG, Kosten T, Woody G. 2012. Opioid detoxification and naltrexone induction strategies: recommendations for clinical practice. Am. J. Drug Alcohol Abuse 38(3):187–99 22404717 10.3109/00952990.2011.653426 PMC4331107

[R150] Skogrand E, Sharpe J, Englander H. 2024. Dispensing methadone at hospital discharge: one hospital’s approach to implementing the “72-hour rule” change. J. Addict. Med 18(1):71–74 37994453 10.1097/ADM.0000000000001246 PMC10873107

[R151] Sofuoglu M, DeVito EE, Carroll KM. 2019. Pharmacological and behavioral treatment of opioid use disorder. Psychiatr. Res. Clin. Pract 1(1):4–15

[R152] Soyka M 2021. Transition from full mu opioid agonists to buprenorphine in opioid dependent patients—a critical review. Front. Pharmacol 12:718811 34887748 10.3389/fphar.2021.718811 PMC8650116

[R153] Soyka M, Franke AG. 2021. Recent advances in the treatment of opioid use disorders—focus on long-acting buprenorphine formulations. World J. Psychiatry 11(9):543–52 34631459 10.5498/wjp.v11.i9.543 PMC8474991

[R154] Spencer M, Garnett M, Miniño A. 2024. Drug overdose deaths in the United States, 2002–2022. NCHS Data Brief 491, Natl. Cent. Health Stat., Hyattsville, MD

[R155] Srivastava AB, Gold MS. 2018. Naltrexone: a history and future directions. Cerebrum 2018:cer-13–18 PMC6353110 30746025

[R156] Stanley TH. 1992. The history and development of the fentanyl series. J. Pain Symptom Manag. 7(3):S3–7 10.1016/0885-3924(92)90047-l 1517629

[R157] Stoeckel H, Hengstmann JH, Schüttler J. 1979. Pharmacokinetics of fentanyl as a possible explanation for recurrence of respiratory depression. Br. J. Anaesth 51(8):741–45 497071 10.1093/bja/51.8.741

[R158] Strain EC, Gaddis A. 2023. Buprenorphine. In The Oxford Handbook of Opioids and Opioid Use Disorder, ed. Dunn KE, pp. 494–520. Oxford, UK: Oxford Univ. Press

[R159] Strang J, Volkow ND, Degenhardt L, Hickman M, Johnson K, 2020. Opioid use disorder. Nat. Rev. Dis. Prim 6(1):3 31919349 10.1038/s41572-019-0137-5

[R160] Strathdee SA, Hallett TB, Bobrova N, Rhodes T, Booth R, 2010. HIV and risk environment for injecting drug users: the past, present, and future. Lancet 376(9737):268–84 20650523 10.1016/S0140-6736(10)60743-X PMC6464374

[R161] Streisand JB, Bailey PL, LeMaire L, Ashburn MA, Tarver SD, 1993. Fentanyl-induced rigidity and unconsciousness in human volunteers: incidence, duration, and plasma concentrations. Anesthesiology 78(4):629–34 8466061 10.1097/00000542-199304000-00003

[R162] Suh Y-G, Cho K-H, Shin D-Y. 1998. Total synthesis of fentanyl. Arch. Pharmacal Res 21(1):70–72 10.1007/BF03216756 9875518

[R163] Tanaka N, Naito T, Yagi T, Doi M, Sato S, Kawakami J. 2014. Impact of CYP3A5^∗^ 3 on plasma exposure and urinary excretion of fentanyl and norfentanyl in the early postsurgical period. Ther. Drug Monit 36(3):345–52 24365989 10.1097/FTD.0000000000000029

[R164] Thakrar AP, Uritsky TJ, Christopher C, Winston A, Ronning K, 2023. Safety and preliminary outcomes of short-acting opioid agonist treatment (sOAT) for hospitalized patients with opioid use disorder. Addict. Sci. Clin. Pract 18(1):13 36829242 10.1186/s13722-023-00368-z PMC9951406

[R165] Tiffany ST, Wray JM. 2012. The clinical significance of drug craving. Ann. N.Y. Acad. Sci 1248(1):1–17 22172057 10.1111/j.1749-6632.2011.06298.x PMC4041083

[R166] Torralva R, Janowsky A. 2019. Noradrenergic mechanisms in fentanyl-mediated rapid death explain failure of naloxone in the opioid crisis. J. Pharmacol. Exp. Ther 371(2):453–75 31492824 10.1124/jpet.119.258566 PMC6863461

[R167] Uddin O, Jenne C, Fox ME, Arakawa K, Keller A, Cramer N. 2021. Divergent profiles of fentanyl withdrawal and associated pain in mice and rats. Pharmacol. Biochem. Behav 200:173077 33316293 10.1016/j.pbb.2020.173077 PMC8065268

[R168] Uhl GR, Koob GF, Cable J. 2019. The neurobiology of addiction. Ann. N.Y. Acad. Sci 1451(1):5–28 30644552 10.1111/nyas.13989 PMC6767400

[R169] Valdez CA, Leif RN, Mayer BP. 2014. An efficient, optimized synthesis of fentanyl and related analogs. PLOS ONE 9(9):e108250 25233364 10.1371/journal.pone.0108250 PMC4169472

[R170] Valentino RJ, Volkow ND. 2018. Untangling the complexity of opioid receptor function. Neuropsychopharmacology 43(13):2514–20 30250308 10.1038/s41386-018-0225-3 PMC6224460

[R171] Varshneya NB, Hassanien SH, Holt MC, Stevens DL, Layle NK, 2022. Respiratory depressant effects of fentanyl analogs are opioid receptor-mediated. Biochem. Pharmacol 195:114805 34673011 10.1016/j.bcp.2021.114805 PMC9022371

[R172] Vearrier D, Grundmann O. 2021. Clinical pharmacology, toxicity, and abuse potential of opioids. J. Clin. Pharmacol 61(Suppl. 2):S70–88 10.1002/jcph.1923 34396552

[R173] Velez FF, Colman S, Kauffman L, Ruetsch C, Anastassopoulos K. 2021. Real-world reduction in healthcare resource utilization following treatment of opioid use disorder with reSET-O, a novel prescription digital therapeutic. Expert Rev. Pharmacoecon. Outcomes Res 21(1):69–76 33146558 10.1080/14737167.2021.1840357

[R174] Vogel M, Hämmig R, Kemter A, Strasser J, Von Bardeleben U, 2016. Use of microdoses for induction of buprenorphine treatment with overlapping full opioid agonist use: the Bernese method. Subst. Abuse Rehabil 7:99–105 27499655 10.2147/SAR.S109919 PMC4959756

[R175] Wakeman SE, Chang Y, Regan S, Yu L, Flood J, 2019. Impact of fentanyl use on buprenorphine treatment retention and opioid abstinence. J. Addict. Med 13(4):253–57 30550392 10.1097/ADM.0000000000000486

[R176] Wakeman SE, Larochelle MR, Ameli O, Chaisson CE, McPheeters JT, 2020. Comparative effectiveness of different treatment pathways for opioid use disorder. JAMA Netw. Open 3(2):e1920622 32022884 10.1001/jamanetworkopen.2019.20622 PMC11143463

[R177] Watts AL, Latzman RD, Boness CL, Kotov R, Keyser-Marcus L, 2023. New approaches to deep phenotyping in addictions. Psychol. Addict. Behav 37(3):361–75 36174150 10.1037/adb0000878 PMC10050231

[R178] Weimer MB, Herring AA, Kawasaki SS, Meyer M, Kleykamp BA, Ramsey KS. 2023. ASAM clinical considerations: buprenorphine treatment of opioid use disorder for individuals using high-potency synthetic opioids. J. Addict. Med 17(6):632–39 37934520 10.1097/ADM.0000000000001202

[R179] Wesson DR, Ling W. 2003. The Clinical Opiate Withdrawal Scale (COWS). J. Psychoact. Drugs 35(2):253–59 10.1080/02791072.2003.10400007 12924748

[R180] Wheeler A-R, Truckenbrod LM, Cooper EM, Betzhold SM, Setlow B, Orsini CA. 2023. Effects of fentanyl self-administration on risk-taking behavior in male rats. Psychopharmacology 240(12):2529–44 37612455 10.1007/s00213-023-06447-y PMC10878692

[R181] Wilde M, Pichini S, Pacifici R, Tagliabracci A, Busardò FP, 2019. Metabolic pathways and potencies of new fentanyl analogs. Front. Pharmacol 10:238 31024296 10.3389/fphar.2019.00238 PMC6461066

[R182] Williams AR, Krawczyk N, Hu M-C, Harpel L, Aydinoglo N, 2023. Retention and critical outcomes among new methadone maintenance patients following extended take-home reforms: a retrospective observational cohort study. Lancet Reg. Health Am 28:100636 38152421 10.1016/j.lana.2023.100636 PMC10751716

[R183] Woolf SH, Schoomaker H. 2019. Life expectancy and mortality rates in the United States, 1959–2017. JAMA 322(20):1996–2016 31769830 10.1001/jama.2019.16932 PMC7146991

[R184] Zacny JP, Lichtor JL, Zaragoza JG, de Wit H. 1992. Subjective and behavioral responses to intravenous fentanyl in healthy volunteers. Psychopharmacology 107(2–3):319–26 1615132 10.1007/BF02245155

[R185] Zangiabadian M, Golmohammadi S, Nejadghaderi SA, Zahmatkesh MM, Nasiri MJ, Sadeghian M. 2022. The effects of naltrexone on retention in treatment and being opioid-free in opioid-dependent people: a systematic review and meta-analysis. Front. Psychiatry 13:1003257 36226100 10.3389/fpsyt.2022.1003257 PMC9548642

[R186] Zibbell JE, Asher AK, Patel RC, Kupronis B, Iqbal K, 2018. Increases in acute hepatitis C virus infection related to a growing opioid epidemic and associated injection drug use, United States, 2004 to 2014. Am. J. Public Health 108(2):175–81 29267061 10.2105/AJPH.2017.304132 PMC5846578

